# Autonomous Non‐Equilibrium Self‐Assembly and Molecular Movements Powered by Electrical Energy**

**DOI:** 10.1002/anie.202214265

**Published:** 2022-12-22

**Authors:** Giulio Ragazzon, Marco Malferrari, Arturo Arduini, Andrea Secchi, Stefania Rapino, Serena Silvi, Alberto Credi

**Affiliations:** ^1^ Institut de Science et d'Ingégnierie Supramoléculaires (ISIS) UMR 7006 University of Strasbourg CNRS 8 allée Gaspard Monge 67000 Strasbourg France; ^2^ Dipartimento di Chimica “Giacomo Ciamician” Università di Bologna via Selmi 2 40126 Bologna Italy; ^3^ Dipartimento di Scienze Chimiche della Vita e della Sostenibilità Ambientale Università di Parma Parco Area delle Scienze 17/A 43124 Parma Italy; ^4^ CLAN-Center for Light-Activated Nanostructures (CLAN) Università di Bologna and Consiglio Nazionale delle Ricerche via Gobetti 101 40129 Bologna Italy; ^5^ Dipartimento di Chimica Industriale “Toso Montanari” Università di Bologna viale del Risorgimento 4 40136 Bologna Italy

**Keywords:** Calixarene, Electrochemistry, Molecular Machines, Non-Equilibrium Processes, SECM

## Abstract

The ability to exploit energy autonomously is one of the hallmarks of life. Mastering such processes in artificial nanosystems can open technological opportunities. In the last decades, light‐ and chemically driven autonomous systems have been developed in relation to conformational motion and self‐assembly, mostly in relation to molecular motors. In contrast, despite electrical energy being an attractive energy source to power nanosystems, its autonomous harnessing has received little attention. Herein we consider an operation mode that allows the autonomous exploitation of electrical energy by a self‐assembling system. Threading and dethreading motions of a pseudorotaxane take place autonomously in solution, powered by the current flowing between the electrodes of a scanning electrochemical microscope. The underlying autonomous energy ratchet mechanism drives the self‐assembly steps away from equilibrium with a higher energy efficiency compared to other autonomous systems. The strategy is general and might be extended to other redox‐driven systems.

## Graphical abstract

By controlling the potential of two electrodes simultaneously, a strategy has been implemented that uses electrical energy to drive self‐assembling systems away from equilibrium. The proposed approach has been demonstrated by modulating the self‐assembly of a calix[6]arene host with a viologen guest, using a scanning electrochemical microscope setup. High thermodynamic efficiencies are obtained.

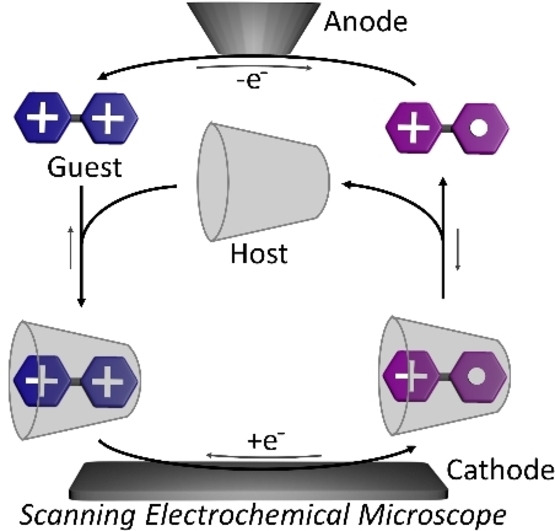

## Introduction

**Figure 1:**
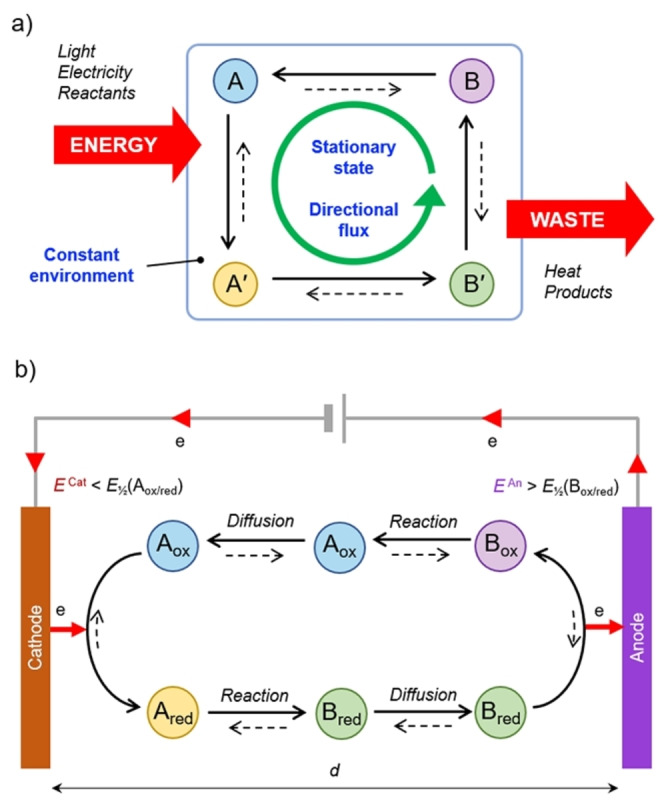
Autonomous chemical cycling sustained by an energy source. a) The concept of autonomous operation of a system described as a cycle of chemical reactions. By exploiting an energy source, the chemical reaction cycle proceeds with a preferential direction, leading to a mass flux. The system, under constant environmental conditions, reaches a non‐equilibrium stationary state that is maintained until energy is supplied. In the case of a molecular machine, at least some of the transformations between A, A′, B, and B′ are molecular movements. Low‐energy products and/or heat are also generated as a result of energy dissipation. b) General scheme of the electrochemically driven sequence of reactions that lead to autonomous cycling between states A and B, obtained by setting two appropriately spaced (*d*) electrodes at different constant potential values (*E* ^Cat^, *E* ^An^). The current flow from the cathode to the anode implies reduction/oxidation, reaction, and diffusion of the components that are continuously formed in the switching cycle.

Autonomous processes^[1]^—which occur multiple times in the presence of a constant energy supply—are ubiquitous in nature and constitute one of the key aspects of life.^[2]^ For this reason, scientists have attempted to create artificial systems that display autonomous behavior, i.e., operate under constant environmental conditions, harnessing energy from the surrounding environment.[^3^, ^4^, ^5^, ^6^, ^7^, ^8^, ^9^] The minimal form of an autonomous chemical system is composed of a cyclic sequence of reactions that occurs persistently. In such a non‐equilibrium system, energy needs to be supplied to impart a directionality in the reaction network, leading to the emergence of a chemical current (Figure 1a).[^7^, ^10^, ^11^, ^12^] This directional flux becomes particularly important when it is associated with molecular motion, as it occurs in natural and artificial molecular machines.[^13^, ^14^, ^15^]

Biomolecular machines, such as ATP synthase, myosin, and kinesin, work autonomously by consuming chemical energy.^[16]^ In fact, these devices ultimately catalyze the conversion of substrates into products while undergoing structural transformations. Inspired by natural systems, chemists have developed autonomous molecular machines such as light‐[^17^, ^18^, ^19^] and chemically driven[^20^, ^21^] rotary motors, switches,[^22^, ^23^, ^24^, ^25^, ^26^] pumps,[^27^, ^28^] walkers,^[29]^ and actuators.[^30^, ^31^, ^32^]

Despite these achievements, the vast majority of artificial molecular machines reported so far (defined as in ref. [5] and [15]) cannot operate autonomously, and making devices of this kind remains a formidable research challenge.[^15^, ^33^, ^34^] In particular, autonomous electrically driven molecular machines are significantly underdeveloped in comparison with light‐ and chemically driven ones. This fact is striking, considering the relevance of electricity in our society and the ease of producing electrical energy from renewable sources. The sole examples available to date are surface‐mounted single‐molecule rotors, whose operation relies on scanning tunneling microscopy (STM) experiments performed under special conditions (ultrahigh vacuum and very low temperature).[^35^, ^36^] In these experiments, the molecule is part of an electric circuit in which the STM tip and the substrate are kept at different potentials. Therefore, it would be desirable to explore strategies that allow the exploitation of electrical energy autonomously at the ensemble level under ambient conditions. This goal is particularly relevant for self‐assembling systems, whose operation at the single‐molecule level has significant fundamental and technical limitations. Efforts in this direction were recently reported in relation to the self‐assembly of a cysteine derivative, which was modulated by controlling the potential of an electrode in the presence of a homogeneous reducing agent, leading to fibers formation under nonequilibrium conditions.^[37]^

To address the outlined challenge, one possibility is to rely on redox‐active oscillating systems, where the origin of the emergent phenomena is rooted in the kinetic control of redox reactions. Albeit prominent examples have been reported,[^38^, ^39^, ^40^, ^41^] this approach suffers from limited flexibility and it is difficult to generalize since it critically relies on specific kinetic features of the oscillating system. An alternative, more viable option consists in interfacing the molecular machine with two electrodes operating at different potentials (Figure 1b). The use of a bipotentiostat enables the independent control of two electrodes, offering a general expedient to impose a thermodynamic driving force—unattainable using a single electrode—that can be exploited to drive systems away from equilibrium electrochemically. The use of such experimental setup in relation to self‐assembly was pioneered by Huskens and co‐workers, who exploited the simultaneous but spatially distinct oxidation and reduction of a redox‐active guest to create a gradient between the electrodes. Equilibrium self‐assembly of the guest to a monolayer of hosts, resulted in the formation of chemical gradients on a surface.^[42]^ Importantly, in such precedents the self‐assembly process adjusts to the electrogenerated gradient, whereas to shift the concentrations of a self‐assembly away from equilibrium, the self‐assembly equilibrium should participate in the path leading to energy dissipation, as illustrated in detail in relation to the exploitation of chemical energy (see Supporting Information Section 1 for an extended discussion of how this situation differs from adaptation to a gradient).^[11]^

Herein, we demonstrate autonomous threading and dethreading movements associated with the self‐assembly away from equilibrium of a pseudorotaxane, leveraging a ratchet mechanism powered by electrical energy. As a result of coupling a self‐assembly reaction to the inter‐electrode current, the assembly steps participate in a reaction cycle characterized by broken detailed balance, in which the electric current is exploited to sustain a non‐equilibrium distribution of assembling species (see Supporting Information Section 1). We envisioned that the inclusion of self‐assembling steps in the energy dissipation path could be demonstrated by means of two electrodes, using a scanning electrochemical microscope (SECM) in a bipotentiostatic configuration. Such a setup enables a careful, simultaneous, and independent control of the electrode potentials and their relative distance, which enables the spatiotemporal coupling between reaction and diffusion processes (Figure 1b), essential to obtain non‐equilibrium steady‐states, thus autonomous behaviour. The use of an ultramicroelectrode (UME), and the capability to position it with submicrometric precision with respect to a second electrode surface, enable the investigation of the oxidation‐reduction processes occurring at both the UME and the biased substrate.[^43^, ^44^] These features, crucial to the present investigation, have been employed to study several molecular systems and materials, also based on square reaction schemes comprising chemical and electrochemical steps and collection‐generation setups, e.g. to study covalent dimerizations.[^44^, ^45^, ^46^, ^47^, ^48^] Yet, to the best of our knowledge, they were never exploited to control a self‐assembly process or molecular movements away from equilibrium.

## Results and Discussion

**Figure 2:**
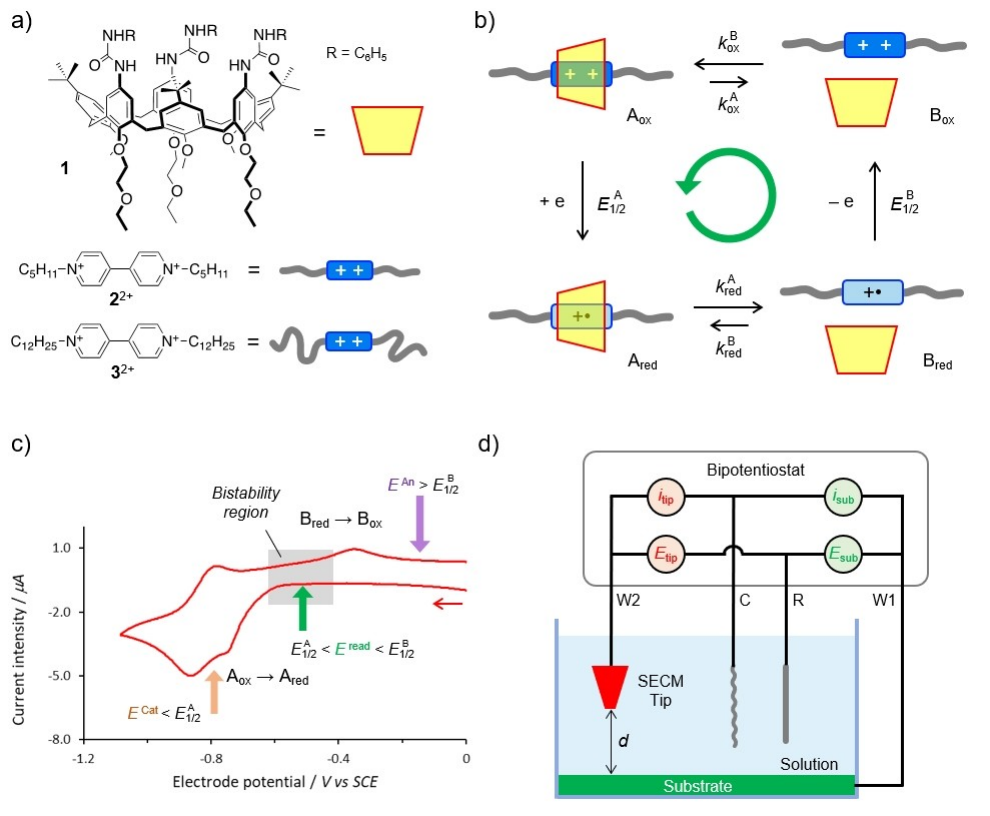
General properties and setup of the investigated system. a) Chemical formulas of the molecular components; tosylate salts of **2** ^2+^ and **3** ^2+^ were employed. b) Network of the self‐assembly and redox reactions that describe the operation of the system. The stability constants of the pseudorotaxanes in the oxidized and reduced states are *K*_ox_=*k* ^B^_ox_/*k* ^A^_ox_ and *K*_red_=*k* ^B^_red_/*k* ^A^_red_, with *K*_ox_≫*K*_red_. c) Relevant features of a typical cyclic voltammetric curve recorded on a 0.58 mM solution of **1** and **2** ^2+^. Experimental conditions: Argon‐purged CH_2_Cl_2_, room temperature, TBAPF_6_ 57 mM, scan rate 200 mV s^−1^. The labels are consistent with those employed in Figure 1b. The reversible process at potential values more negative than ca. −0.8 V corresponds to the reduction of free **2** ^+^⋅ to **2**. d) Scheme of the scanning electrochemical microscope (SECM) with bipotentiostatic configuration. The circuit allows the simultaneous and independent control of the potential of the two working electrodes (W1, substrate, and W2, tip). The distance between the substrate and the tip (*d*) can be easily varied by employing the SECM stepper motors and piezoelectrical components. C and R are the counter and reference electrodes, respectively. The solution is contained in a deoxygenated and sealed electrochemical cell.

To test our hypothesis, we focused on an electroactive pseudorotaxane, [**1⊃2**]^2+^, composed by tris(N‐phenylureido)calix[6]arene **1** and 1,10‐dipentyl‐4,4′‐bipyridinium **2** ^2+^ (Figure 2a). Indeed, **1** is a well‐known host for the latter compound and analogous axles; the pseudorotaxane formed with **2** ^2+^ has a stability constant *K*_o*x*_=5.0×10^6^ M^−1^ in CH_2_Cl_2_.^[49]^ The assembly and disassembly of these components can be controlled electrochemically, according to the square scheme reported in Figure 2b.^[50]^ Specifically, the reduction of [**1⊃2**]^2+^ to [**1⊃2**]^+^⋅ results in the complete dethreading of **2** ^+^⋅. This process can be induced and monitored in cyclic voltammetry (CV) experiments (Figure 2c), where a reduction current is observed for potential values more negative than ca. −0.7 V vs SCE, indicative of the reduction of [**1⊃2**]^2+^. In the return scan, the observation of an oxidation current uniquely at ca. −0.3 V—a potential typical of the oxidation of free **2** ^+^⋅ to **2** ^2+^—highlights the complete dethreading of **2** ^+^⋅ from **1** on the CV time scale, in line with spectroscopic evidence.^[51]^

At potential values more positive than −0.3 V both **2** ^2+^ and [**1⊃2**]^2+^ are thermodynamically stable, whereas at values more negative than −0.7 V the reduced forms **2** ^+^⋅ and [**1⊃2**]^+^⋅ are stable. At intermediate potentials, i.e., in the range between −0.4 and −0.6 V, **2** ^+^⋅ and [**1⊃2**]^2+^ are the thermodynamically stable species. Therefore, in agreement with the concept presented in Figure 1b, the autonomous threading and dethreading of **2** ^2+^ and **1** can occur in the following scenario. The cathode is kept at −0.8 V, a potential at which [**1⊃2**]^2+^ is reduced to [**1⊃2**]^+^⋅. Owing to the fast dethreading of the latter and the diffusion time from cathode to anode (which we can control by changing the inter‐electrode distance *d*), free **2** ^+^⋅ arrives at the anode, maintained at 0 V, where it is promptly reoxidized to **2** ^2+^. Eventually, rethreading of the latter into **1** regenerates the [**1⊃2**]^2+^ complex (which is stable near the anode) on a time scale faster than diffusion of free **2** ^2+^ to the cathode. The pseudorotaxane can then reach the cathode by diffusion, thereby closing the autonomous threading‐dethreading cycle, which comprises the self‐assembly steps.

### Experimental design and setup

Our SECM setup (Figure 2d) allows to control the potential of a substrate electrode (*E*_sub_) and that of the probe electrode (*E*_tip_) simultaneously, and vary them in a programmed fashion. We will refer to the probe electrode (a platinum UME disk) as the tip. For example, *E*_sub_ can be set at a constant value, while scanning *E*_tip_ to perform a CV using the tip as the working electrode, thus allowing the detection of the species that has formed at the substrate and has diffused to the tip. The distance between the tip and the substrate (*d*) is controlled by measuring the tip current while approaching the substrate (probe approach curves, see Supporting Information Section 2). In our experimental setup this distance is set at 25 μm, unless otherwise stated.

Other important elements for the present discussion are indeed the diffusion rates, and the threading and dethreading rates. In particular, the autonomous mechanism described above requires that the dethreading of electrogenerated [**1⊃2**]^+^⋅ (i.e., A_red_→B_red_ in Figure 1b) be faster than its diffusion to the anode. Similarly, the rethreading of **2** ^2+^ produced at the anode (i.e., B_ox_→A_ox_ in Figure 1b) should occur before free **2** ^2+^ reaches the cathode. On the other hand, the species formed upon the redox process that occurs at one electrode must diffuse to the other electrode fast enough to be detected on the time scale of the electrochemical experiment. The values of the diffusion coefficient (*D*) of **2** ^2+^ and [**1⊃2**]^2+^, determined by voltammetric measurements under the same conditions used in the SECM experiments (see Supporting Information Sections 4 and 5), are respectively 9.6×10^−6^ and 6.5×10^−6^ cm^2^ s^−1^, and are reasonably assumed to be equal to those of the corresponding reduced forms, **2** ^+^⋅ and [**1⊃2**]^+^⋅.^[52]^ From the expression of the mean square displacement, one can thus estimate that an inter‐electrode distance *d* of 25 μm is covered in about 3–400 ms. The threading rate constant, determined by stopped‐flow absorption experiments in CH_2_Cl_2_ with a 100‐fold excess of tetrabutylammonium hexafluorophosphate (TBAPF_6_), resulted to be *k* ^B^_ox_=2.3×10^6^ M^−1^ s^−1^ (see Supporting Information Section 3). Such rate implies that at the concentrations employed in the SECM experiments the threading half‐life of **1** and **2** ^2+^ is 3 ms, much faster than the inter‐electrode diffusion time. A similar conclusion holds for the dethreading of [**1⊃2**]^+^⋅ (see Supporting Information Section 6).

These analyses illustrate that (i) the redox reaction performed at one electrode is rapidly followed by a chemical transformation to restore equilibrium, and (ii) the species generated in the process can be promptly detected at the other electrode. Hence, the selected pseudorotaxane appears to fulfill the requirements for electrically driven autonomous operation in a SECM setup.

### Demonstration of Autonomous Operation

**Figure 3:**
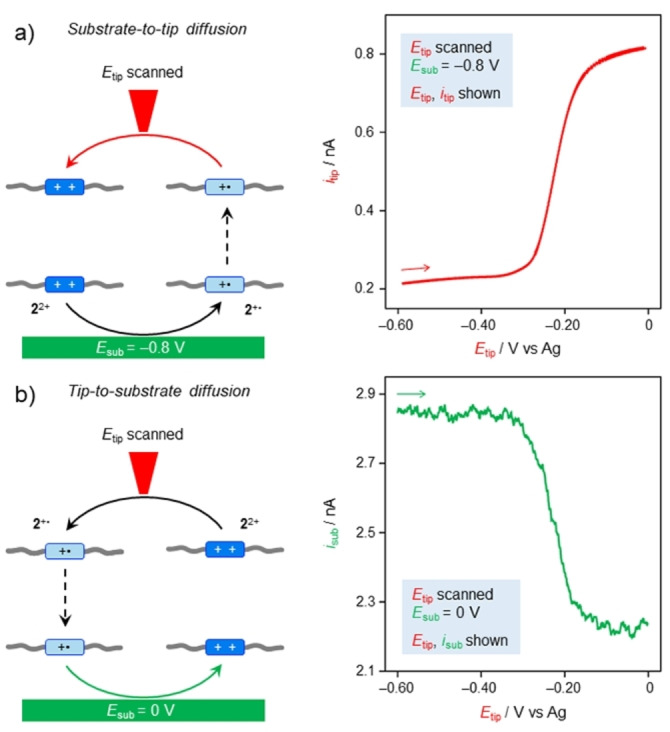
Electrogeneration of species at one electrode and detection at the other electrode. a) A CV recorded at the tip electrode detects the presence of **2** ^+^⋅ formed upon reduction of **2** ^2+^ at −0.8 V at the substrate electrode and diffused to the tip. b) The positive current recorded at the substrate electrode reveals the oxidation of **2** ^+^⋅ produced during a CV scan at the tip. Note that oxidation and reduction currents have a positive and negative sign, respectively. Inter‐electrode diffusion is represented by dashed arrows, while the colored arrows indicate the electron‐transfer processes monitored in the correspondingly colored voltammetric curve. Experimental conditions: CH_2_Cl_2_, r.t., **2** ^2+^ 240 μM, TBAPF_6_ 24 mM, 100 mV s^−1^, *d*=25 μm, potential values are referenced to an Ag pseudo‐reference electrode.

SECM is commonly used to probe with the UME the processes occurring at the substrate electrode. Yet, the fact that the species produced at one electrode reach the other electrode was also confirmed under the currently employed experimental conditions. To prove that the species produced at the substrate electrode reach the tip electrode, a solution of **2** ^2+^ was initially analyzed. In particular, a CV was performed at the tip electrode, while the substrate was kept at a fixed potential of −0.8 V, a potential at which the stable species is **2** ^+^⋅ (Figure 3a). The CV scan performed at the tip reveals an oxidation process (positive current) coherent with the re‐oxidation of **2** ^+^⋅ (Figure 3a, red curve), indicating that the species produced at the substrate electrode arrive at the tip under the employed conditions.

We also confirmed that the species produced at the tip electrode reach the substrate electrode. To this aim, the latter was set at 0 V, and its current was monitored while the tip potential was scanned between 0 and −0.6 V, thus reducing **2** ^2+^ to **2** ^+^⋅ (Figure 3b). During the reduction scan at the tip, an oxidative current was recorded at the substrate, confirming that the species produced at the tip—in this case **2** ^+^⋅—arrive at the substrate electrode, where re‐oxidation to **2** ^2+^ takes place (Figure 3b, green curve). Having established that the two electrodes are sufficiently near to allow the species produced at the electrode surface to diffuse from one electrode to the other, we proceeded demonstrating that the threading and dethreading processes occur faster than diffusion to the other electrode.

**Figure 4:**
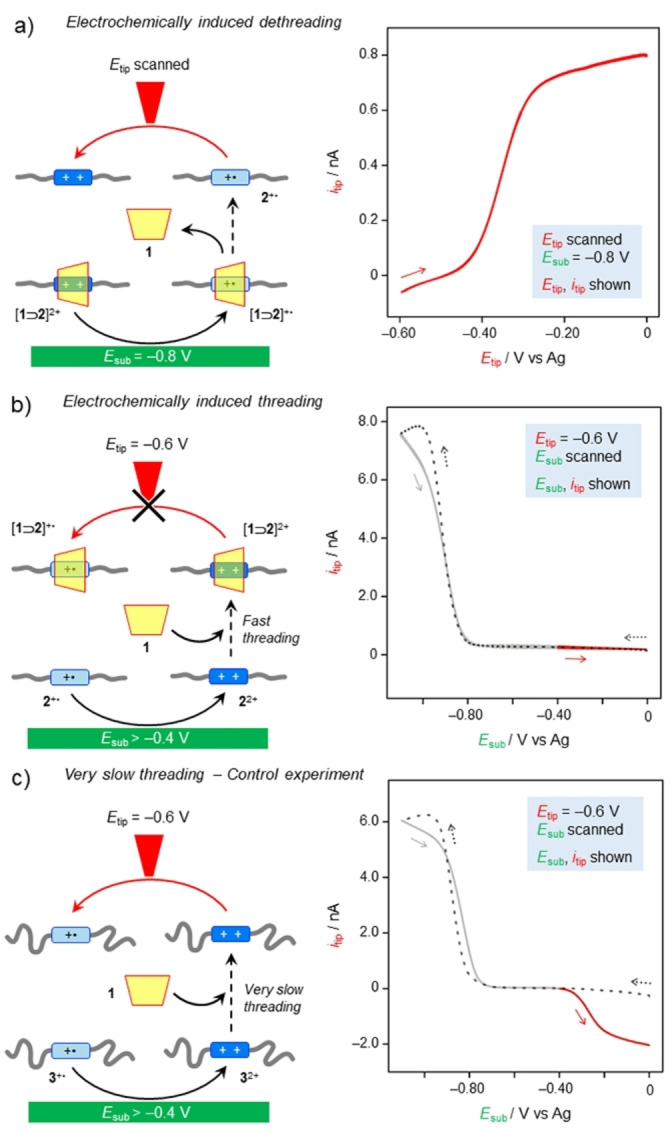
Demonstration of electrochemically induced threading‐dethreading and subsequent inter‐electrode diffusion. a) A CV scan performed at the tip electrode detects **2** ^+^⋅ formed at the substrate electrode upon reduction of [**1⊃2**]^2+^ and subsequent dethreading. b) Free **2** ^2+^, generated from **2** ^+^⋅ during a CV scan at the substrate electrode, quickly rethreads with **1** on the diffusion time scale; the resulting [**1⊃2**]^2+^ complex is not detected at the tip because it is electrochemically silent at −0.6 V. c) The same experiment in (b) performed with guest **3** ^2+^ in the place of **2** ^2+^. The former is characterized by much slower threading kinetics than the latter; as a result, free **3** ^2+^ produced at the substrate electrode does not undergo significant threading on the diffusion time scale, thus it is reduced at the tip. Note that the oxidation and reduction currents have a positive and negative sign, respectively. Inter‐electrode diffusion is represented by dashed arrows, while the colored arrows indicate the electron‐transfer processes monitored in the correspondingly colored voltammetric curve. For clarity, the forward (cathodic) scan in (b) and (c) is represented with a dotted line. Experimental conditions: CH_2_Cl_2_, r.t., **1** 380 μM, **2** ^2+^ or **3** ^2+^ 240 μM, TBAPF_6_ 24 mM, 100 mV s^−1^, *d*=25 (a) or <4 (b, c) μm, potential values are referenced to an Ag pseudo‐reference electrode.

Specifically, regarding the dethreading process, it has to be demonstrated that following reduction of the pseudorotaxane [**1⊃2**]^2+^, dethreading occurs before the reduced pseudorotaxane reaches the tip. In this scenario, when the pseudorotaxane is reduced at the substrate electrode, the tip detects the free, reduced axle **2** ^+^⋅ and not the reduced pseudorotaxane. In order to prove this sequence of events, a solution of **1** : **2** ^2+^=1.6 : 1 was investigated. Under this condition, the majority of axle **2** ^2+^ is present in the form of pseudorotaxane [**1⊃2**]^2+^. As shown in Figure 4a, the substrate was kept at −0.8 V, a potential at which the reduced pseudorotaxane [**1⊃2**]^+^⋅ is produced. Simultaneously, a CV was performed at the tip electrode, scanning between −0.6 and 0 V. This CV scan is aimed at detecting the free axle **2** ^+^⋅; an oxidative current was indeed recorded at −0.3 V (Figure 4a, red curve), i.e., a potential much closer to the one for oxidation of free **2** ^+^⋅ (−0.25 V) rather than that of the pseudorotaxane (−0.8 V). This observation indicates that the reduction‐induced dethreading of **2** ^+^⋅ occurs faster than diffusion to the other electrode.

To complete the requirements for autonomous operation, it has to be demonstrated that self‐assembly reaction participate to the chemical reaction cycle that is driven away from equilibrium, this condition implies that upon re‐oxidation of **2** ^+^⋅ to **2** ^2+^, threading is faster than diffusion to the opposite electrode. Gaining direct evidence of this process is far from trivial because, in principle, one should detect a newly formed pseudorotaxane [**1⊃2**]^2+^ in a solution that contains a bulk amount of the same species. However, we reasoned that, in simple CV reduction experiments, during the return (anodic) scan, the uncomplexed guest **2** ^+^⋅ is detected, as evidenced by an oxidation wave occurring at a potential of ca. −0.3 V (Figure 2c). Therefore, when the potential of the substrate electrode is scanned, uncomplexed **2** ^2+^ is generated during the return scan at potentials more positive than ca. −0.3 V. To assess whether the newly formed species reaches the tip electrode before or after forming the thermodynamically more stable pseudorotaxane [**1⊃2**]^2+^, *E*_tip_ was kept at −0.6 V, a value at which the free axle **2** ^2+^ can be reduced, but not the [**1⊃2**]^2+^ complex (Figure 4b). Indeed, no reduction current was observed under these conditions (Figure 4b, red trace), suggesting that rethreading occurs faster than diffusion of free **2** ^2+^ towards the tip electrode.

Importantly, the same experiment was performed using axle **3** ^2+^, i.e., an axle analog of **2** ^2+^ bearing dodecyl alkyl chains instead of pentyl ones (Figure 1a), which impart a much slower threading rate with **1** (*k* ^B^_ox_=2.6×10^2^ M^−1^ s^−1^) compared with parent **2** ^2+^ (*k* ^B^_ox_=2.3×10^6^ M^−1^ s^−1^).^[49]^ In this case, a reduction current was detected at the tip potential (Figure 4c, red trace). This observation is coherent with **3** ^2+^ reaching the tip electrode before rethreading, thus corroborating the above interpretation. This control experiment also highlights the effect of the self‐assembly/disassembly dynamics on the electrochemical response of the system. To verify our analysis, we have performed numerical simulations using the experimentally determined values for redox processes, self‐assembly reactions and diffusion coefficients. All the data presented in Figure 3 and 4, as well as macroscopic CV were reproduced, supporting our interpretation (see Supporting Information Section 8, Figures S13–S19).

**Figure 5:**
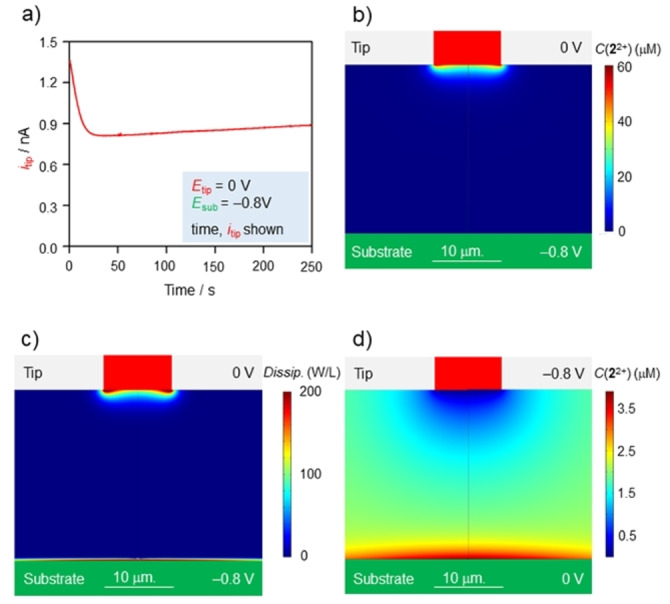
Autonomous electrochemical operation. a) Current recorded at the tip electrode in an amperometric experiment performed by fixing the tip and substrate potentials at 0 and −0.8 V, respectively. b) Simulated concentration of **2** ^2+^ at *t*=150 s. c) Simulated total dissipation (power per unit volume) associated with self‐assembly reactions at *t*=150 s. d) Simulated concentration of **2** ^2+^ at *t*=150 s, with *E*_tip_=−0.8 V, *E*_sub_=0 V. Experimental conditions: CH_2_Cl_2_, r.t., **1** 380 μM, **2** ^2+^ 240 μM, TBAPF_6_ 24 mM, 100 mV s^−1^, *d*=25 μm, *E*_tip_=0 V and *E*_sub_=−0.8 V (a–c) or *E*_tip_=−0.8 V and *E*_sub_=0 V (d), potential values are referenced to an Ag pseudo‐reference electrode.

Overall, we have shown that the tip‐substrate distance is sufficiently small to ensure the mutual communication of electroactive species, while large enough to ensure that the self‐assembly reactions occur before reaching the opposite electrode by diffusion. Hence, an experiment can be performed to drive the self‐assembly processes away from equilibrium using electrical energy as depicted in Figure 1b. The substrate and tip electrodes were positioned at *d*=25 μm, their potentials were set respectively at −0.8 and 0 V, and the tip current was monitored for a period of 250 s (Figure 5a). A quasi‐stationary current of 0.85 nA was measured, indicating that redox reactions occurred at the two electrodes.

Numeric simulations could reproduce the experimental observations (see Supporting Information Section 8, Figures S13–S19) and revealed the dynamic features of the observed non‐equilibrium steady‐state. Concentration maps indicate that **2** ^2+^ is formed at the tip and readily depleted in its vicinity to form [**1⊃2**]^2+^, which can diffuse unperturbed to the substrate (Figure 5b and Supporting Information Section 8, Figures S20, S21). Analogously, [**1⊃2**]^+^⋅ is formed at the substrate and immediately disassembles. The net rates of self‐assembly of [**1⊃2**]^2+^ and [**1⊃2**]^+^⋅, i.e., chemical currents, quantitatively report on these processes. Their values change depending on the specific point in space that is considered, but are equal and opposite to 3.4 fmol s^−1^ when integrated over the entire volume comprised between the electrodes, i.e., the portion of space perturbed by the imposed non‐equilibrium conditions (see Supporting Information Section 9). Comparison of this rate with the electric current reveals that, on average, 1.4 electrons transferred from the cathode to the anode cause one dethreading and one rethreading events. As a consequence, axles in proximity of the electrodes perform a threading‐dethreading cycle every 26 seconds; this means that, on average, any given species can undergo about 10 threading‐dethreading events on the experimentally observed timescale (250 s).

Access to the dynamics of the systems allowed us to evaluate the efficiency at which the input energy is exploited. In the context of autonomous systems, the energy efficiency is the amount of input energy that is absorbed by the chemical reactions coupled to the energy source. In the present case the coupled reactions are the self‐assembling steps, and the input energy is used (absorbed) to sustain a non‐equilibrium distribution of species.^[53]^ Therefore, the efficiency discussed herein does not coincide with the collection efficiency typically discussed in SECM experiments.^[54]^ This quantity corresponds to the energy dissipated in the self‐assembly steps, which can be retrieved from the rates of assembly and disassembly and visualized (Figure 5c and Supporting Information Section 9 for detailed mathematical treatment). Dissipation takes place in close proximity to both electrodes while the central part of the solution remains close to equilibrium. In our experimental setup most of the energy input (*W*_in_=*n* *F*Δ*E* with *n*, number of exchanged electrons; *F*, Faraday constant; Δ*E*, electrode potential difference) is associated with the current flowing between the substrate and counter electrodes. Comparing this energy input with the energy dissipated in the self‐assembly steps affords an efficiency of 2.3×10^−6^. While important, this value should be reconsidered when comparing the efficiency of the present systems with those of chemically or light‐driven autonomous artificial molecular motors that were described in detail from the thermodynamic point of view.[^53^, ^55^] Indeed, thermodynamic analysis of said motors does not take into account the background energy loss. For a chemically driven motor this energy loss is associated with the background decomposition of the chemical energy source, and for a light‐driven system to light absorbed without inducing photochemical reactions. Here, the unproductive background energy loss is associated with the current flowing between substrate and counter electrodes. Therefore, for a proper comparison only the charge flux between the tip and the substrate mediated by the self‐assembling species should be taken into account. Considering only this contribute, the resulting energy efficiency is 0.06, which is higher than those of other autonomous systems reported to date for which the same analysis is available (Table S7).[^20^, ^27^, ^53^, ^55^, ^56^] The large discrepancy between the two efficiency values points at the importance of the experimental setup in determining the thermodynamic efficiency of autonomous electrically driven systems.

To validate the potential of our system in exploring multiple non‐equilibrium steady states, we have operated our system also under opposite boundary conditions, i.e., *E*_tip_=−0.8 and *E*_sub_=0 V, (see Supporting Information Section 7, 8, Figures S10, S22, S23) proving that multiple non‐equilibrium steady‐states are experimentally accessible using this system.[^55^, ^57^] While the recorded current (−0.8 nA) is similar to the one measured under opposite boundary conditions, the distribution of species is completely different. This fact is evident when comparing the stationary concentrations of **2** ^2+^ in the two cases (cf. Figure 5b and d). In this different operation mode the calculated efficiency is even higher, reaching 0.09.

Since it is known that thermodynamic stabilities dictate the directionality in systems operating according to an energy ratchet mechanism, but not in those relying on information ratchet, we simulated the autonomous operation of an identical system in which the thermodynamic stabilities of oxidized and reduced pseudorotaxanes were inverted (see Supporting Information Section 9).^[12]^ As a result, inversion of the self‐assembly current was also observed, validating the operation of this autonomous non‐equilibrium system as an autonomous energy ratchet.

## Conclusion

In summary, we have investigated the autonomous non‐equilibrium self‐assembly of an electrically driven system, a pseudorotaxane performing threading and dethreading motions. Mechanical steps are part of a cyclic network of chemical reactions that occurs directionally under non‐equilibrium conditions, operating as a chemical engine,^[58]^ according to the terminology proposed by Leigh and co‐workers. As for other autonomous systems, the autonomous operation was established through a series of individually provable premises.^[20]^ The present system operates according to an energy ratchet mechanism, which so far could operate autonomously only when driven by light irradiation. Here, spatial separation^[59]^ of the oxidation and reduction reaction, coupled to diffusion,^[60]^ enables an autonomous chemically driven energy ratchet mechanism. Therefore, the system operation mode differs from other autonomous molecular machines.[^17^, ^20^] As a consequence, high efficiencies are obtained, up to 0.09 in the optimal operation setup. Theoretical aspects of network requirements and the effect on the efficiency of changing parameters are currently under investigation and will be the subject of a later publication. The strategy may be generalized and up‐scaled, but in this case it should leverage mass‐transport mechanisms other than diffusion, which is effective only over short distances; moreover, it might be applied to other redox‐driven nanomachines, including molecular pumps^[61]^ able to perform work repetitively.[^62^, ^63^] Besides molecular machines, the same strategy might be used to operate non‐equilibrium supramolecular systems that rely on electrical energy, such as fiber‐forming monomers.[^37^, ^41^] Implementing the autonomous use of electrical energy by supramolecular assemblies to perform molecular motions advances our ability to control matter away from equilibrium.[^9^, ^64^]

## Conflict of interest

The authors declare no conflict of interest.

1

## Supporting information

As a service to our authors and readers, this journal provides supporting information supplied by the authors. Such materials are peer reviewed and may be re‐organized for online delivery, but are not copy‐edited or typeset. Technical support issues arising from supporting information (other than missing files) should be addressed to the authors.

Supporting Information

## Data Availability

The data that support the findings of this study are available in the Supporting Information of this article.
