# Supplementary material for: Autonomous Non‐Equilibrium Self‐Assembly and Molecular Movements Powered by Electrical Energy
Source: Angew Chem Int Ed Engl. 2022 Dec 22;62(5):e202214265. doi: 10.1002/anie.202214265 (PMC10107654; doi:10.1002/anie.202214265)
Supplement: Supplementary file 1 — Supporting Information [file ANIE-62-0-s001.pdf]

Angewandte Chemie
Eine Zeitschrift der Gesellschaft Deutscher Chemiker

Supporting Information

Autonomous Non-Equilibrium Self-Assembly and Molecular Movements Powered by Electrical Energy

G. Ragazzon, M. Malferrari, A. Arduini, A. Secchi, S. Rapino*, S. Silvi*, A. Credi*

Table of contents:

1. Difference from adaptation to a gradient 3

2. Materials and methods 5

Reagents and solvents

UV-Visible spectra and kinetic experiments

Conventional voltammetric experiments

Scanning electrochemical experiments

3. UV-Visible spectra and kinetic experiments 7

4. Conventional voltammetric experiments 8

5. Determination of the diffusion coefficients 11

6. Time scales for diffusion, threading and dethreading 12

Diffusion.

Threading of $\mathbf{2}^{2+}$.

Dethreading of $[\mathbf{1}\supset\mathbf{2}]^{+\bullet}$

Threading of $\mathbf{3}^{2+}$

7. Additional amperometric experiment 13

8. Simulations 14

9. Insights on system performance 24

Dynamic properties

Efficiency

Operation mechanism

10. References 27

1. Difference from adaptation to a gradient

An essential feature of the present work is that the host-guest equilibrium participates in the reaction sequence funneling electrons from one electrode to the other. This coupling results in non-equilibrium populations of host and guest, which are associated with energy absorption from the imposed gradient. This behavior is markedly different from simple adaptation to a gradient in a Le Chatelier-like manner. To clarify this very important difference, here we compare the two cases in greater detail.

<u>Case 1:</u> adaptation according to Le-Chatelier's principle. The self-assembly equilibria do not participate in the reaction cycle.

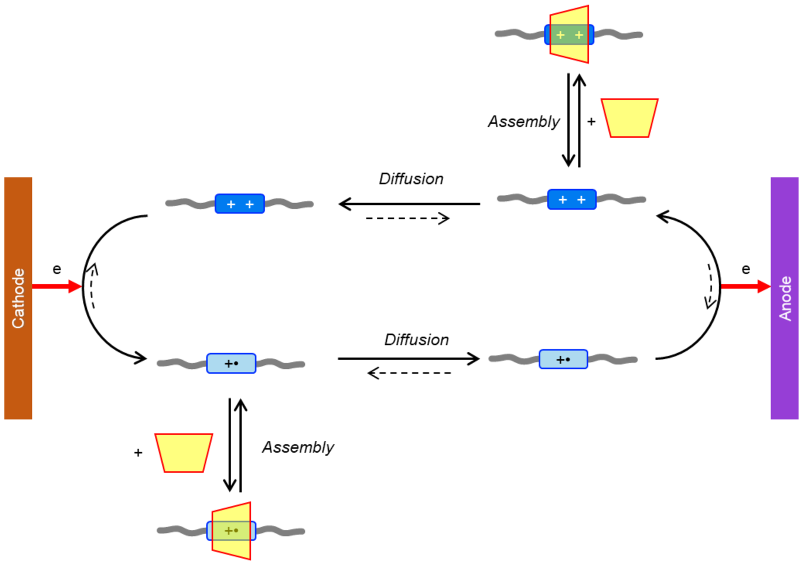

Figure S1. Chemical reaction network associated with a system in which the self-assembly reactions do not participate in the reaction cycle (state of the art).

In case 1, at the steady-state, the self-assembly reactions are at equilibrium. Detailed balance applies to these steps. Since the concentration of oxidized/reduced paraquat follows a gradient in space, the concentration of host-guest complex also follows a gradient in space. In such systems, energy cannot be stored in the self-assembly steps, and the concept of efficiency does not apply. The flow of an electron from the cathode to the anode is not associated with any net reaction (paraquat simply transfers electrons from cathode to anode).

<u>Case 2:</u> non-equilibrium adaptation (this work). The assembly equilibria participate in the reaction cycle.

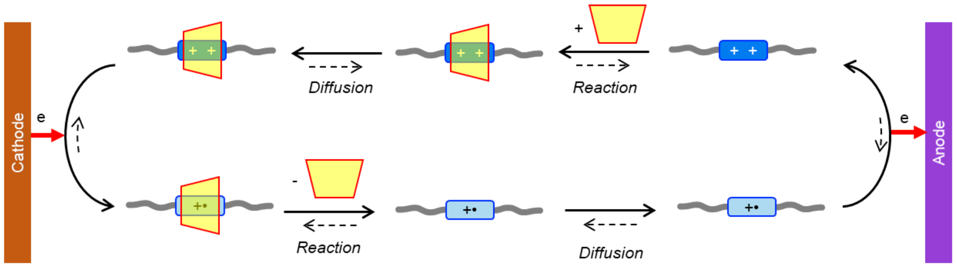

Figure S2. Chemical reaction network associated with a system in which the self-assembly reactions participate in the reaction cycle (this work).

In case 2, at the steady-state the self-assembly reactions are not at equilibrium, because they are part of the cycle. Detailed balance is not respected in the self-assembly steps (on average, oxidized paraquat assembles and the reduced host-guest complex disassembles). Therefore, the concentration of host-guest species is not merely adapting to the concentration gradient of oxidized/reduced paraquat. In such a system, part of the energy provided by the electric current is harvested to afford a biased distribution of species (the assembly reactions not being at equilibrium). Contrary to case 1, now the flow of an electron from the cathode to the anode is coupled with a chemical reaction, specifically a disassembly event.

2. Materials and methods

Reagents and solvents. Compounds **1**, **2**$(TsO)_2$ and **3**$(TsO)_2$ were available from previous investigations, where a detailed NMR, MS, UV-vis, and electrochemical characterization is available.[1] Dichloromethane used as the solvent was anhydrous > 99.8%, purchased from Sigma Aldrich and was used as received. Tetrabutylammonium hexafluorophosphate and ferrocene were purchased from Aldrich; the latter was purified by sublimation.

UV-Visible spectra and kinetic experiments. Absorption spectra collected prior to kinetic studies were recorded on a Cary 300 (Varian - Agilent) spectrophotometer, using a dedicated tandem cuvette, which comprises two compartments separated by a wall with a hole for the mixing (optical path length of each compartment 0.44 cm). Such a cuvette enables a direct comparison of the sum of the spectra of two components with the spectrum of the mixture of the same components (see e.g. Figure S4), thereby allowing the detection of minimal spectral changes upon mixing. Threading kinetics were investigated on air-equilibrated solutions thermostated at 20° C using a stopped-flow spectrophotometer equipped with a 1 cm pathlength cell and a driving ram for the mixing system at the N_2 pressure of 8.5 bar. The data obtained were fitted with software SPECFIT[2] with a a kinetic model comprising a second-order forward and a first-order backward reactions, with the constrain that the ratio between the two constants should equal the thermodynamic constant.

Conventional voltammetric experiments. Cyclic voltammetry (CV) and differential pulse voltammetry (DPV) experiments with conventional microelectrodes were carried out in argon-purged CH_2Cl_2 with an Autolab 30 multipurpose instrument interfaced to a PC. The working electrode was a Pt electrode (Amel, diameter 1 mm) carefully polished with an alumina-water slurry on a felt surface, immediately before use. A Pt wire, separated from the solution by a frit, was employed as counter electrode, whereas an Ag wire was used as a quasi-reference electrode. Ferrocene was added as an internal standard. The supporting electrolyte was added in a 100-fold proportion with respect to the sample concentration. Cyclic voltammograms were obtained at sweep rates varying from 0.05 to 5 V s^{-1}. Differential pulse voltammograms were recorded with a rate of 0.02 V s^{-1} and a peak height of 0.075 V. The IR compensation implemented within the software was employed to minimize the resistance of the solution. In any instance, the full electrochemical reversibility of the voltammetric wave of ferrocene was taken as an indicator of the absence of uncompensated resistance effects. Samples could be investigated for at least a full day without noticing any sign of fatigue, provided the rigorous exclusion of oxygen.

Scanning electrochemical experiments. Scanning electrochemical microscopy (SECM) studies were carried out with a CHI910B SECM bipotentiostat from CH Instruments Inc. (Austin, Texas). Platinum microelectrode (Sensolytics GmbH Pt 10 μm diameter, RG 10) was employed as working electrodes and probe of the SECM; a 2 mm Pt disk electrode (CH Instruments, Inc.) served as substrate electrode. The Pt substrate electrode was mounted at the bottom of a Teflon cell. Both probe and substrate electrodes were carefully polished with diamonded paper and sonicated in a bath sonicator just before the use. A Pt wire was employed as counter electrode and a Ag wire served as a quasi-reference electrode. A silicone bulb was used to shield the electrochemical cell from atmospheric oxygen. The oxygen was removed from the solution by Ar bubbling. To position the probe at micrometric distances from the substrate, we performed probe approach curves following the reduction of the studied pseudorotaxanes or compound **2**$(TSO)_2$, according to the analyzed solution. In Figure S3 is reported a representative approach curve performed to position the tip. The current signals were normalized to the iT,∞ value, namely the bulk current estimated applying the same bias as for the approach curves at distances longer than 500 μm. The tip-substrate separation is reported as $L=d/r_{tip}$, where d is the tip-substrate distance and r_{tip} is the active radius of the tip. To estimate the precise position of the tip with respect to the substrate, approach curves were fitted by Cornut- Lefrou equations for purely positive feedback.[3] MIRA software by Prof. Wittstock (university of Oldenburg, DE) was used for fitting all current approach curves and for

plotting the images. The CVs reported in the main text were recorded at tip-substrate distances in the range of 4-25 μm. The amperometric measurements, reported in the following and conducted to estimate the efficiency of the process, were conducted at about 25 μm tip-substrate separation. Small variations (of few μm) of the tip-substrate distance, in the aforementioned range, would not affect the observed qualitative behavior and eventually only the magnitude of recorded currents would be influenced. The use of ultramicroelectrodes guarantees for small capacitive currents at the tip and increases the temporal resolution and the sensitivity of the employed method. For this reason, the discussed amperometric experiments take into consideration the current registered at the tip electrode. Simulations of SECM experiments were performed using COMSOL Multiphysics 5.5 available on campus.[4,5]

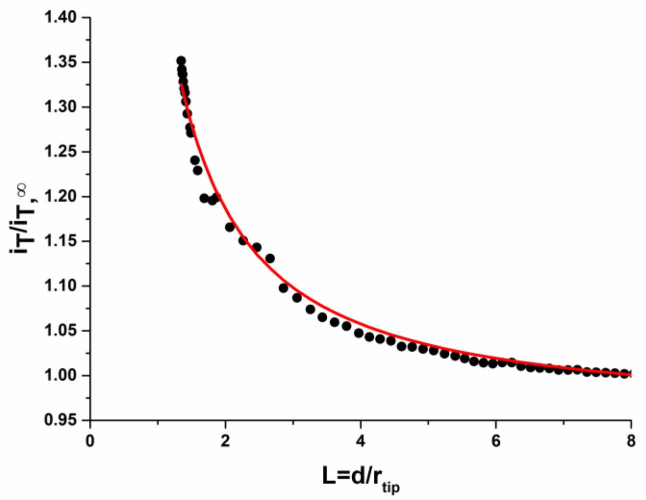

Figure S3. Approach curve on the Pt substrate electrode (black points), in red is reported the fitted curve. Probe working electrode Pt, 10 μm diameter, $C(\mathbf{2}^{2+})$ = 240 μM, E_{tip}=-0.4 V. Experimental conditions: CH_2Cl_2, room temperature, >100-fold $TBAPF_6$.

3. UV-Visible spectra and kinetic experiments

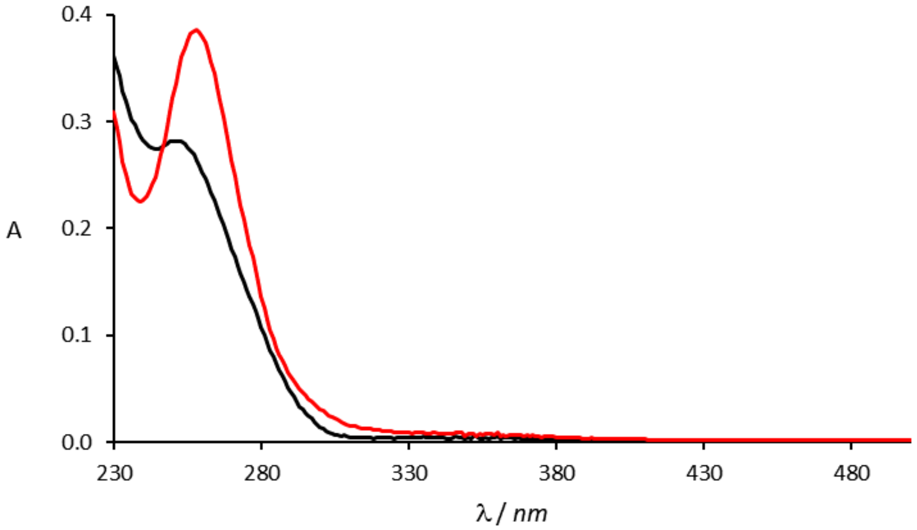

Figure S4. Comparison of the sum of the spectra **1** and $\mathbf{2}^{2+}$ (black line) with the spectrum of the mixture of the same components (red line) obtained with the tandem cuvette (*vide supra*). Concentrations before mixing: $C(\mathbf{1})$ = 10.1 μM, $C(\mathbf{2}^{2+})$ = 10.7 μM. Experimental conditions: CH_2Cl_2, room temperature, 100-fold $TBAPF_6$.

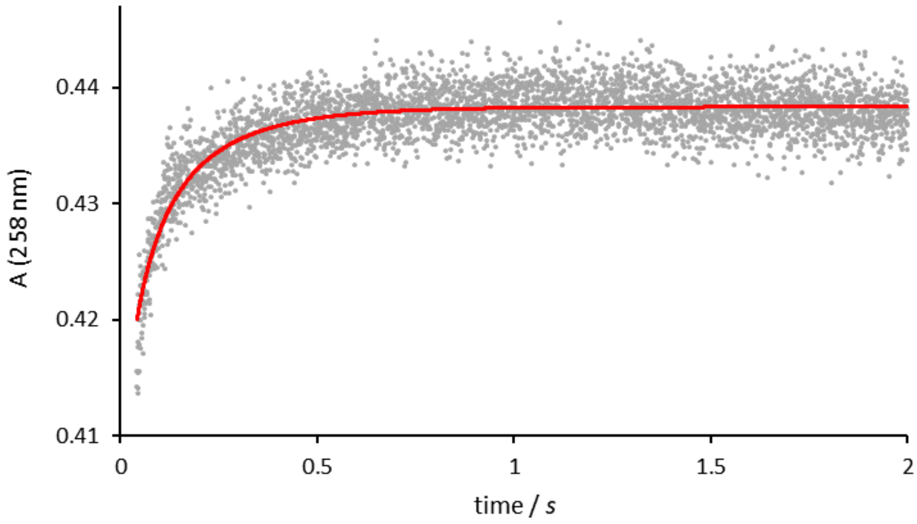

Figure S5. Time-dependent absorption changes at 258 nm (grey dots) observed upon mixing of **1** (5.05 μM) and $\mathbf{2}^{2+}$ (5.35 μM). The red line is the best fit to a kinetic model comprising a second-order forward and a first-order backward reactions. Experimental conditions: CH_2Cl_2, 293 K, 100-fold $TBAPF_6$.

4. Conventional voltammetric experiments

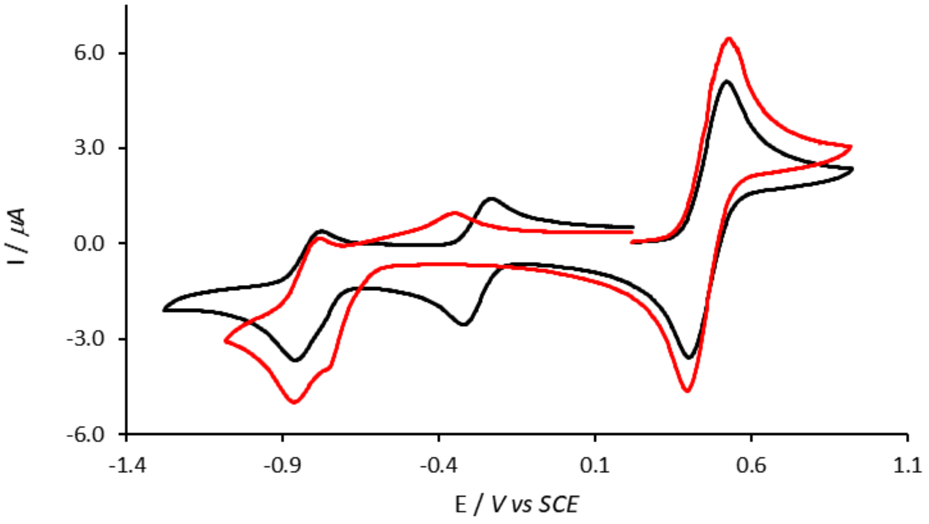

Figure S6. Cyclic voltammetry of a 0.58 mM solution of **2**$^{2+}$ (black line) and of **2**$^{2+}$ in presence of 1 equivalent of **1** (red line). Experimental conditions: Argon purged CH_2Cl_2, room temperature, $TBAPF_6$ 57 mM, scan rate 200 mV s^{-1}. The process at +0.460 V is the oxidation of ferrocene, used as internal standard.

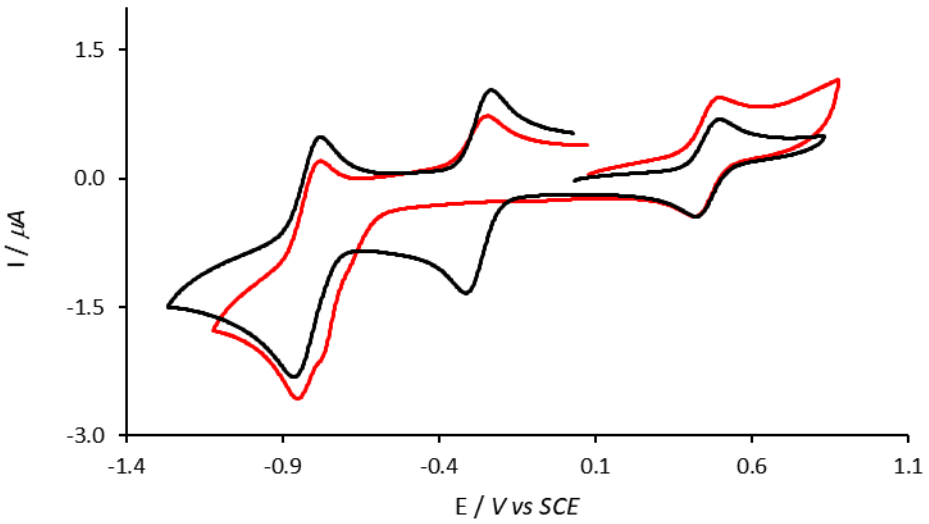

Figure S7. Cyclic voltammetry of a 0.25 mM solution of **3**$^{2+}$ (black line) and of **3**$^{2+}$ in presence of 1.5 equivalents of **1** (red line). Experimental conditions: Argon purged CH_2Cl_2, room temperature, $TBAPF_6$ 30 mM, scan rate 200 mV s^{-1}. The process at +0.460 V is the oxidation of ferrocene, used as internal standard.

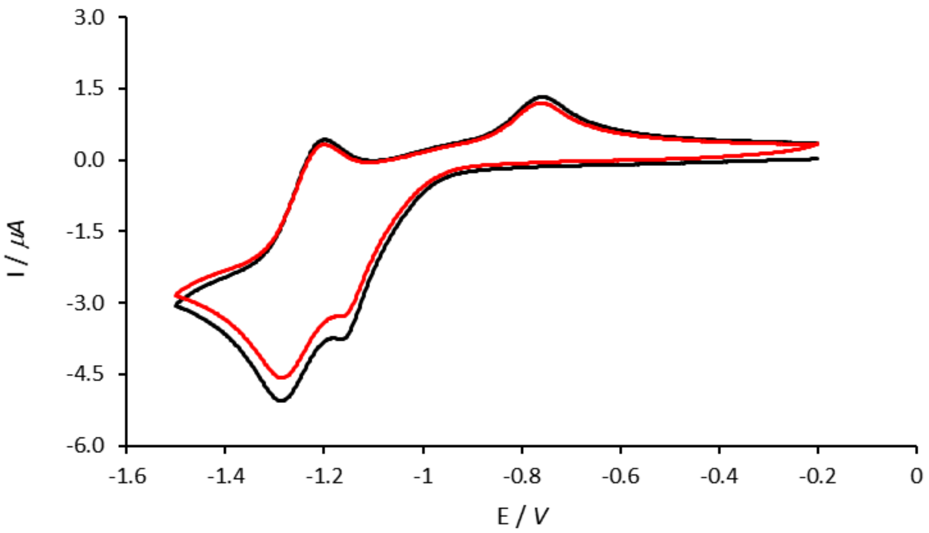

Figure S8. Cyclic voltammetries of a 0.58 mM solution of **2**$^{2+}$ in presence of 1 equivalent of **1**. Black line: first cycle; red line: second cycle, performed without renewing the diffusion layer. Experimental conditions: Argon purged CH_2Cl_2, room temperature, $TBAPF_6$ 57 mM, scan rate 200 mV s^{-1}.

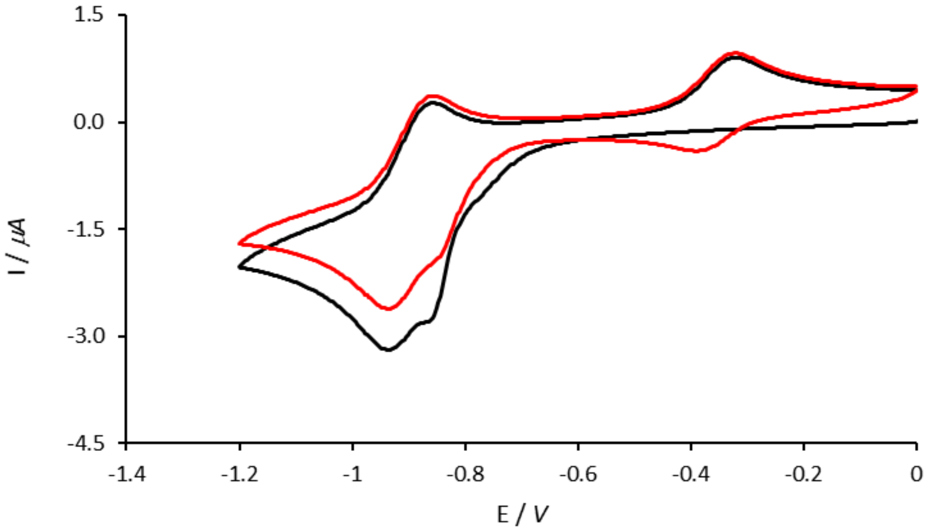

Figure S9. Cyclic voltammetries of a 0.24 mM solution of **3**$^{2+}$ in presence of 1.5 equivalents of **1**. Black line: first cycle; red line: second cycle, performed without renewing the diffusion layer. Experimental conditions: Argon purged CH_2Cl_2, room temperature, $TBAPF_6$ 30 mM, scan rate 200 mV s^{-1}.

Table S1. Reduction potential values of the examined compounds versus the saturated calomel electrode (SCE).

Compound	E_1 (V vs SCE)	E_2 (V vs SCE)
$\mathbf{2}^{2+}$	–0.28	–0.81
$[\mathbf{1}\supset\mathbf{2}]^{2+}$	[a]	–0.81
$\mathbf{3}^{2+}$	–0.28	–0.83
$[\mathbf{1}\supset\mathbf{3}]^{2+}$	[a]	–0.82

[a] Irreversible process

5. Determination of the diffusion coefficients

The diffusion coefficients of **2**$^{2+}$ and **3**$^{2+}$ were determined by means of cyclic voltammetric experiments, applying the Randles–Ševčík equation 1:[6]

$$i = 0.4463 \times n \times F \times A \times C \times \sqrt{\left(\frac{n \times F \times D \times v}{R \times T}\right)} \quad (1)$$

where *i* is the peak current, *n* is the number of exchanged electrons, *F* is the Faraday constant, *A* is the electrode area, *C* and *D* are respectively the concentration and the diffusion coefficient of the electroactive species, *v* is the scan rate, *R* is the gas constant and *T* is the temperature.

Cyclic voltammetries of the two compounds were recorded at different scan rates, ranging from 0.05 to 0.2 V s^{-1}, in the presence of ferrocene. The concentration of both the examined compounds and ferrocene were known. From the ratios of the peak currents, by rearranging equation 1, the diffusion coefficients *D* of compounds **2**$^{2+}$ and **3**$^{2+}$ were calculated as follows:

$$D = D(Fc) \times \left(\frac{C(Fc) \times i}{C \times i(Fc)}\right)^2 \quad (2)$$

where *D*(Fc) is the diffusion coefficient of ferrocene (2.3×10^{-5} cm^2 s^{-1}),[7] *C*(Fc) and *C* are the concentrations of ferrocene and of either **2**$^{2+}$ or **3**$^{2+}$, respectively, and *i*(Fc) and *i* are the peak currents of ferrocene and of either **2**$^{2+}$ or **3**$^{2+}$, respectively.

The diffusion coefficients of the pseudorotaxane complexes [**1**⊃**2**]$^{2+}$ and [**1**⊃**3**]$^{2+}$ were determined from the diffusion coefficients of the related axle molecules **2**$^{2+}$ and **3**$^{2+}$, respectively, using the following correlation[8,9] between molecular weight (*MW*) and diffusion coefficient (*D*):

$$\frac{D1}{D2} = \sqrt[3]{\frac{MW2}{MW1}} \quad (3)$$

Values of diffusion coefficients are gathered in Table S2.

Table S2. Molecular weight and diffusion coefficient of the examined compounds.

Compound	*MW* (g mol^{-1})	*D* (cm^2 s^{-1})
**2**$(TsO)_2$	641	$9.6 \cdot 10^{-6}$ [a]
**3**$(TsO)_2$	837	$7.7 \cdot 10^{-6}$ [a]
[**1**⊃**2**]$(TsO)_2$	2107	$6.5 \cdot 10^{-6}$ [b]
[**1**⊃**3**]$(TsO)_2$	2303	$5.5 \cdot 10^{-6}$ [b]

[a] Measured from cyclic voltammetry (see the text for details).
[b] Calculated with equation 3.

6. Time scales for diffusion, threading and dethreading

Diffusion. For a one-dimensional diffusion process, the mean square displacement $<x>^2$ is related to the diffusion coefficient D and diffusion time τ_D by the formula:

$<x>^2 = 2\, D\, \tau_D$ (4)

Thus, on the basis of the diffusion coefficients reported in Table S2, the time periods necessary for the examined compounds to cover an inter-electrode distance d = 250 μm = $2.5 \cdot 10^{-3}$ cm are:

$\mathbf{2}^{2+}$: $\tau_D = d^2 / 2D(\mathbf{2}^{2+}) = (2.5 \cdot 10^{-3})^2/(2 \times 9.6 \cdot 10^{-6}) = 0.326$ s = 326 ms (5)

$\mathbf{3}^{2+}$: $\tau_D = (2.5 \cdot 10^{-3})^2/(2 \times 7.7 \cdot 10^{-6}) = 0.406$ s = 406 ms (6)

$[\mathbf{1} \supset \mathbf{2}]^{2+}$: $\tau_D = (2.5 \cdot 10^{-3})^2/(2 \times 6.5 \cdot 10^{-6}) = 0.481$ s = 481 ms (7)

$[\mathbf{1} \supset \mathbf{3}]^{2+}$: $\tau_D = (2.5 \cdot 10^{-3})^2/(2 \times 5.5 \cdot 10^{-6}) = 0.568$ s = 568 ms (8)

Threading of $\mathbf{2}^{2+}$. Under the employed experimental conditions, the threading process follows second-order kinetics and the half-life of $\mathbf{2}^{2+}$ can be calculated as follows:

$t_{1/2} = 1 / (k^B_{ox} \times C_0)$ (9)

where k^B_{ox} is the rate constant for the self-assembly of $[\mathbf{1} \supset \mathbf{2}]^{2+}$ and C_0 is the initial concentration of calixarene **1**. As $k^B_{ox} = 2.3 \cdot 10^6$ M^{-1} s^{-1} and $C_0 = 1.4 \cdot 10^{-4}$ M, the half-life of the free axle calculated from equation 9 is $t_{1/2}(\mathbf{2}^{2+})$ = 3 ms.

Since $t_{1/2}(\mathbf{2}^{2+}) << \tau_D(\mathbf{2}^{2+})$ (equation 5), the (re)-threading of **1** and $\mathbf{2}^{2+}$ generated at the anode is complete during the diffusion to the opposite electrode. Thus, the species that reaches the cathode is $[\mathbf{1} \supset \mathbf{2}]^{2+}$.

Dethreading of $[\mathbf{1} \supset \mathbf{2}]^{+\bullet}$. Treating the dethreading of the reduced pseudorotaxane as a first-order process, and considering that the dethreading rate constant was estimated to be[10] $k^A_{red} = 2 \cdot 10^6$ s^{-1}, the corresponding half-life is:

$t_{1/2}([\mathbf{1} \supset \mathbf{2}]^{+\bullet}) = \ln 2 / (k^A_{red}) = 3.5 \cdot 10^{-7}$ s = 0.35 μs (10)

Since the half-life of the reduced pseudorotaxane is much shorter than its inter-electrode diffusion time, complete dethreading of the $[\mathbf{1} \supset \mathbf{2}]^{+\bullet}$ complex generated at the cathode takes place during diffusion. Therefore, the species that reaches the anode is free $\mathbf{2}^{+\bullet}$.

Threading of $\mathbf{3}^{2+}$. The second-order threading rate constant of **1** and $\mathbf{3}^{2+}$ is $k^B_{ox} = 2.6 \cdot 10^2$ M^{-1} s^{-1}.[1] Therefore, the half-life of $\mathbf{3}^{2+}$ under the conditions employed is:

$t_{1/2}(\mathbf{3}^{2+}) = 1 / (k^B_{ox} \times C_0) = 27.5$ s (11)

Since $t_{1/2}(\mathbf{3}^{2+}) >> \tau_D(\mathbf{3}^{2+})$ (equation 6), no appreciable threading of the $\mathbf{3}^{2+}$ axle generated at the anode occurs during the inter-electrode diffusion. The species that reaches the cathode is free $\mathbf{3}^{2+}$.

7. Additional amperometric experiment

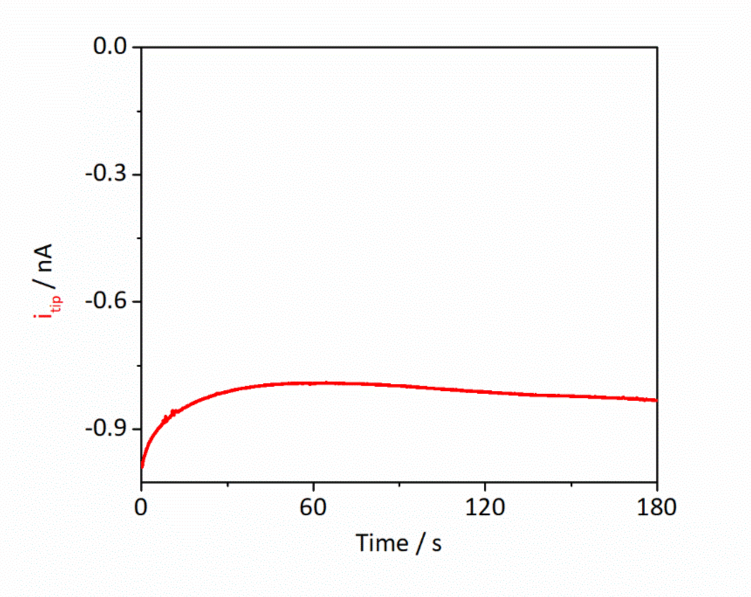

Figure S10. Amperometric experiment in which E_{sub} = 0 V and E_{tip} = –0.8 V. Conditions: C(**1**) = 380 μM, C(**2**$^{2+}$) = 240 μM, d = 25 μm.

8. Simulations

The simulated chemical reaction network is reported in Figure S11.

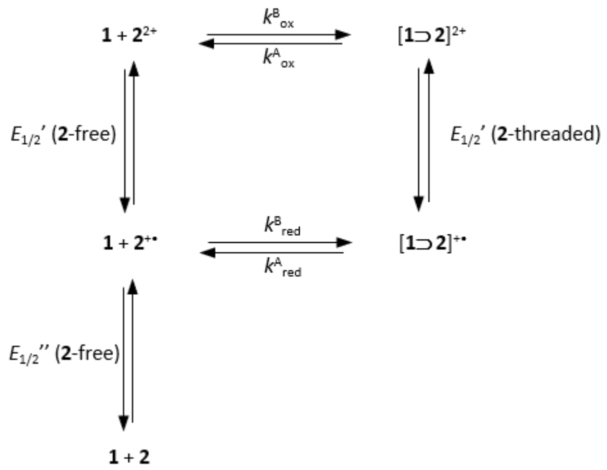

Figure S11. Simulated chemical reaction network with employed notation. The same network was employed to simulate experiments performed using **3**$^{2+}$.

Electrochemical reactions were simulated according to Butler-Volmer equation, employing a transfer coefficient equal to 0.5 in all cases. All cyclic voltammetry simulations were performed at a scan rate of 100 mV s^{-1}. The parameters employed are collected in Table S3 and S4. These parameters are very similar to those employed to fit cyclic voltammetry experiments reported in ref. 10, and are coherent with quantitative disassembly of the investigated pseudorotaxanes, in line with spectroscopic evidence reported by some of us in ref. 11.

Table S3. Electrochemical parameters employed to perform numeric simulations.

Name	Value (units)	Description
$E_{1/2}$' (**2**-free)	–0.28 (V)[a]	Reduction potential
$E_{1/2}$'' (**2**-free)	–0.81 (V)[a]	Reduction potential
$E_{1/2}$' (**2**-threaded)	–0.70 (V)[b]	Reduction potential
$E_{1/2}$' (**3**-free)	–0.28 (V)[a]	Reduction potential
$E_{1/2}$'' (**3**-free)	–0.83 (V)[a]	Reduction potential
$E_{1/2}$' (**3**-threaded)	–0.70 (V)[c]	Reduction potential
*i*0 (**2**)	964850 (A m^{-2})[b]	Reference exchange current for free **2**[d]
*i*0 ([**1**⊃**2**])	5790 (A m^{-2})[b]	Reference exchange current for threaded **2**[d]

[a] Experimentally determined, see Table S1.
[b] From ref. 10.
[c] Assumed identical to $E_{1/2}$' (**2**-threaded).
[d] Identical reference exchange currents were employed for reactions involving **3**$^{2+}$.

Chemical reactions were described according to mass-action kinetics, ensuring that the employed rate constants respected the constraints imposed by microscopic reversibility. The parameters employed are collected in Tables S4.

Table S4. Parameters associated with self-assembly reactions employed to perform numeric simulations.

Constant	Value (units)
k^{B}_{ox} (**2**)	2.3×10^{3} (m^3 mol^{-1} s^{-1})[a]
k^{A}_{ox} (**2**)	2.9 (s^{-1})[b]
k^{B}_{red} (**2**)	1.3×10^{2} (m^3 mol^{-1} s^{-1})[c]
k^{A}_{red} (**2**)	2.0×10^{6} (s^{-1})[d]
k^{B}_{ox} (**3**)	2.6×10^{-1} (m^3 mol^{-1} s^{-1})[e]
k^{A}_{ox} (**3**)	3.3×10^{-4} (s^{-1})[f]
k^{B}_{red} (**3**)	1.4×10^{-2} (m^3 mol^{-1} s^{-1})[c]
k^{A}_{red} (**3**)	2.3×10^{2} (s^{-1})[f]

[a] Experimentally determined, see main text and Figure S5.
[b] Determined according to $k^{A}_{ox} = k^{B}_{ox} / K_{ox}$, using the experimentally determined value of $K_{ox} = 8.0\times10^{2}$ mM^{-1} obtained under the same experimental conditions, in the presence of excess $TBAPF_6$.[10]
[c] Imposed by microscopic reversibility.
[d] From ref. 10.
[e] From ref. 1.
[f] Obtained from the corresponding value for compound **2**, multiplied by k^{B}_{ox} (**3**) / k^{B}_{ox} (**2**) = 1.13×10^{-4}.

In simulations performed in the absence of **1**, the bulk and boundary concentration of $\mathbf{2}^{2+}$ was initially set to 240 μM. In the presence of **1**, the following concentrations were employed: **1**, 142 μM; $\mathbf{2}^{2+}$, 2 μM; $[\mathbf{1}\supset\mathbf{2}]^{2+}$, 238 μM, which reflect the equilibrium populations of these species. The same initial values were employed in simulations regarding compound $\mathbf{3}^{2+}$. The concentration of all other species was set to 0 μM. All time-dependent simulations were preceded by a steady-state study, which was used to refine the bulk concentrations of all species, which were then employed in time-dependent simulations. In the case of amperometric experiments the starting concentrations were calculated performing the steady-state study by fixing the substrate potential at the target value and disabling the tip electrode. Boundary concentrations were kept fixed at the above indicated values.

Diffusion coefficients reported in Table S2 were used. The reduction coefficient of reduced species was reasonably assumed identical to the one of the parent oxidized species.[12]

The geometry was constructed using a 2D axial symmetry, based on the parameters reported in Table S5, employed to construct the polygon described by the points indicated in Table S6 and depicted in Figure S12. 25 μm was employed as typical tip-substrate operating distance (*d*), while *d* = 4 μm was employed to simulate the contact distance.

Table S5. Geometric parameters employed to perform numeric simulations.

Name	Value (μm)	Description
r_sub_boundary	1250	Total model radius
r_sub_e	1000	Substrate electrode radius
r_tip_e	5	Tip electrode radius
r_tip_tot	50	Total tip radius
r_tip_upper	500	Geometric parameter
h_boundary	500	Geometric parameter
h_tip	h_tip_sub + 300	Geometric parameter
h_tip_sub	25 or 4	Tip-substrate distance (*d*)

Table S6. Coordinates of the points used to construct model geometry.

x-coordinate	y-coordinate
0	0
r_tip_tot	0
r_sub_e	0
r_sub_boundary	0
r_sub_boundary	h_boundary
r_tip_upper	h_boundary
r_tip_upper	h_tip
r_tip_tot	h_tip_sub
r_tip_e	h_tip_sub
0	h_tip_sub

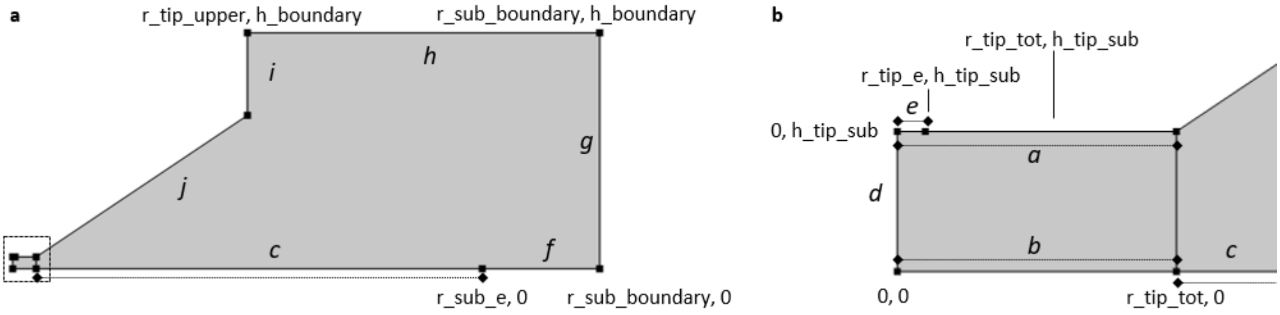

Figure S12. (a) Geometry employed in the present simulations for *d* = 25 μm and (b) magnification of the tip electrode area, indicated by the dashed square in panel (a). Relevant point coordinates and segments are indicated, with lines drawn to avoid ambiguity in the identification of selected segments.

It was confirmed that further extending the dimension of the substrate electrode does not change the results of simulations. Indeed, for a species having a diffusion coefficient of 1.0×10^{-5} cm^2 s^{-1} it takes about 500 s to cover 1 mm by diffusion, which is longer that any time scale explored in this model.

The mesh was constructed using a triangular user-controlled mesh setup, with minimum element size of r_tip_e/100, and a maximum element growth rate of 1.1. The following specific constraints were imposed:

- At point (r_tip_e, h_tip_sub), a maximum element size of r_tip_e/50, decreased to r_tip_e/100 when calculating dissipation.
- Along segments *a* and *b* in Figure S12, a maximum element size of r_tip_e/10, decreased to r_tip_e/50 when calculating dissipation.
- Along segment *c* in Figure S12, a maximum element size of 2×r_tip_e.
- In the domain comprised between the tip and the substrate, *i.e.* the rectangle with dimensions r_tip_tot, h_tip_sub, a maximum element size of r_tip_e/3.
- When calculating dissipation, a maximum element size of r_tip/50 was imposed along the substrate electrode boundary, up to 75 μm from the origin, i.e., for additional 25 μm beyond segment *b* end. For the data presented in Figure S24b, a maximum element size of r_tip/200 was imposed along the tip electrode surface.

It was confirmed that further increasing the mesh density at the electrode surfaces and along the symmetry axis did not change simulation outcomes.

Axial symmetry was imposed along segment *d* in Figure S12. Segment *e* served as tip electrode, while segments *b* and *c* served as substrate electrode. Bulk concentrations were imposed along segments *g* and *h*., e.g. the concentration of $[\mathbf{1}\supset\mathbf{2}]^{2+}$ is fixed at 238 μM, which is its equilibrium concentration (*vide supra*). Segments *f, i* and *j* were imposed as insulating surfaces.

The appropriateness of the parameters employed was confirmed by simulating the cyclic voltammetry of mixtures of **1** and $\mathbf{2}^{2+}$ or $\mathbf{3}^{2+}$ at the substrate electrode (after increasing the tip-substrate distance to 200 μm). The results are reported in Figures S13 and S14, and reproduce the behavior observed in Figures S8 and S9, with differences in current values due to the different size of the electrodes.

The slight differences in current observed between numeric simulations and experiments can be ascribed to the following main factors. (1) In the experiments, the real concentration can be higher that the nominal one, because purging with an inert gas the solution to eliminate O_2 results in dichloromethane evaporation, leading to an increase in solution concentration; this effect is consistent with simulated currents being slightly smaller than experimental values. (2) In the experiments, potentials are controlled with respect to a quasi-reference electrode, thus electrode potential may slightly differ from the nominal values. We have confirmed that by adjusting the concentration within the experimental error it is possible to precisely reproduce the experimental current values. Herein, we report simulation obtained employing the nominal values for concentrations and electrode potentials. (3) Amperometric simulations were performed following a pre-equilibration step (vide supra) which includes the experimental response of the substrate electrode. Instead, the experimental response of the substrate electrode reflects in a slower approach of the current to its asymptotic value. This process follows a Cottrel-like evolution, accounting for the observed induction time of ca. 20 s. Throughout the manuscript, amperometric experiments have been discussed only considering the quasi steady-state properties of the system, which follow the induction time.

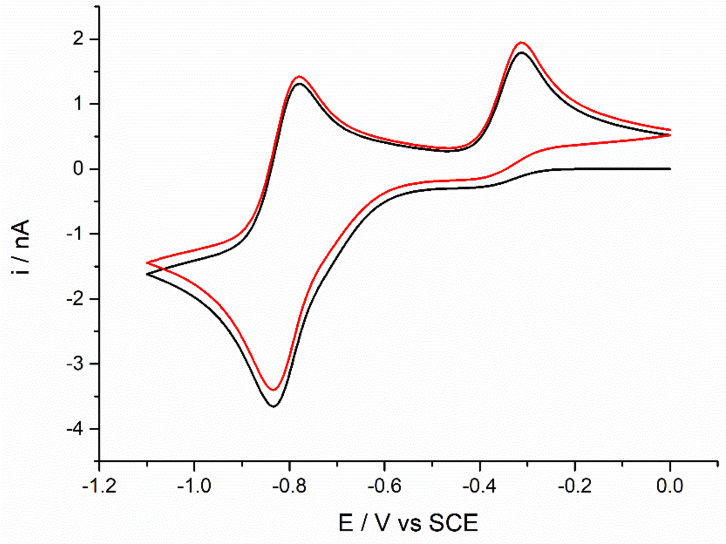

Figure S13. Simulated cyclic voltammetries of a 0.24 mM solution of $\mathbf{2}^{2+}$ in presence of 1.5 equivalents of **1**. Black line: first cycle; red line: second cycle, performed without renewing the diffusion layer. Scan rate 200 mV s^{-1}.

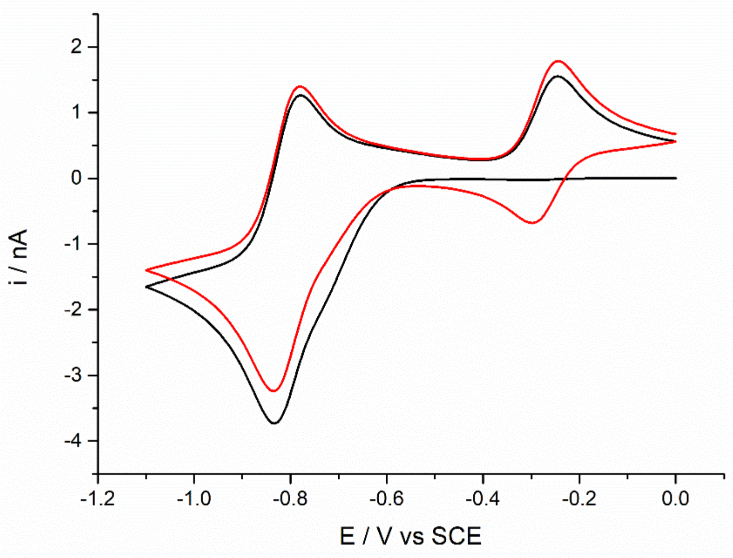

Figure S14. Simulated cyclic voltammetries of a 0.24 mM solution of $\mathbf{3}^{2+}$ in presence of 1.5 equivalents of **1**. Black line: first cycle; red line: second cycle, performed without renewing the diffusion layer. Scan rate 200 mV s^{-1}.

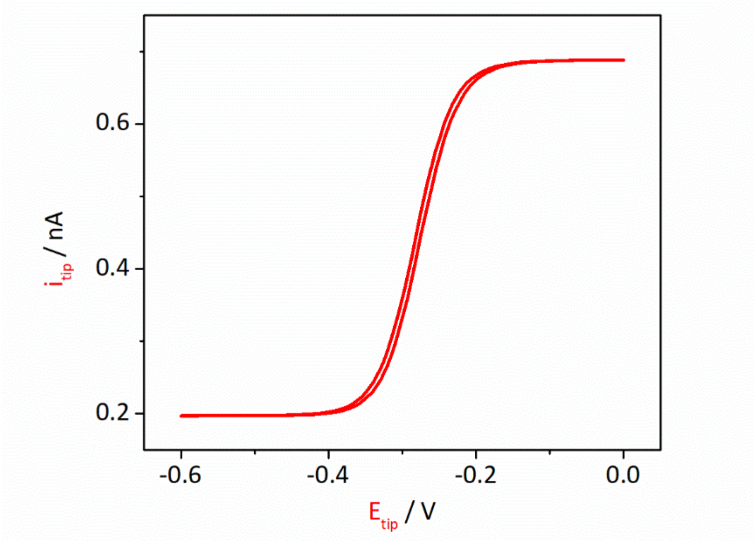

Figure S15. Simulation of experiment shown in Figure 3a. CV performed at the tip electrode while the substrate is kept at a potential of –0.8 V. Conditions: C(**2**$^{2+}$) = 240 μM, d = 25 μm.

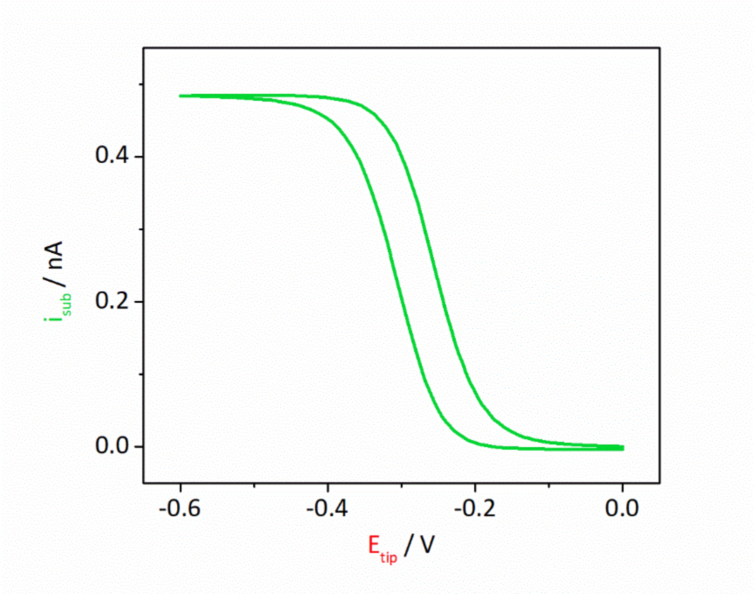

Figure S16. Simulation of experiment shown in Figure 3b. CV performed at the tip electrode while the substrate is kept at a potential of 0 V. Conditions: C(**2**$^{2+}$) = 240 μM, d = 25 μm.

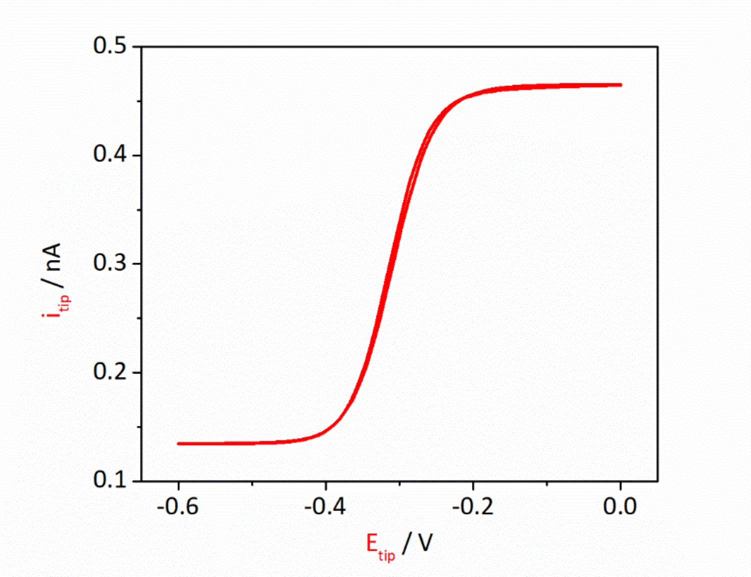

Figure S17. Simulation of experiment shown in Figure 4a. CV performed at the tip electrode while the substrate is kept at a potential of –0.8 V. Conditions: *C*(**1**) = 380 μM, *C*($\mathbf{2}^{2+}$) = 240 μM, *d* = 25 μm.

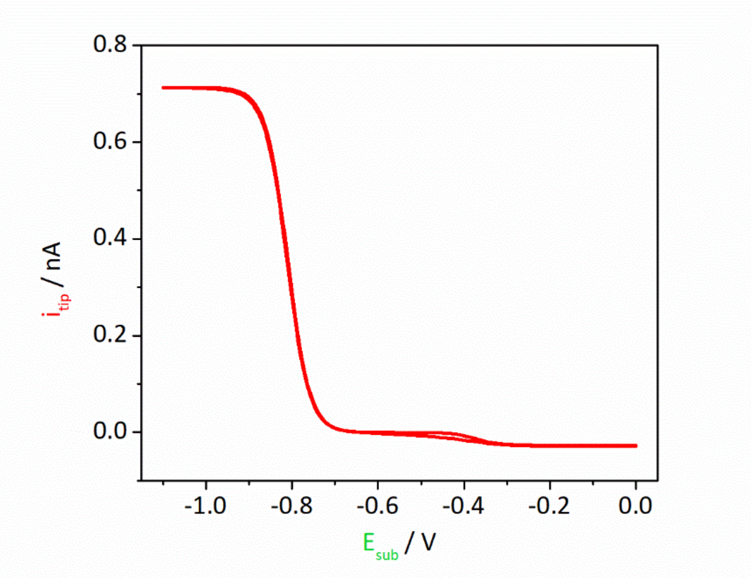

Figure S18. Simulation of experiment shown in Figure 4b. CV performed at the substrate electrode while E_{tip} is kept at a potential of –0.6 V. Conditions: *C*(**1**) = 380 μM, *C*($\mathbf{2}^{2+}$) = 240 μM, *d* = 4 μm.

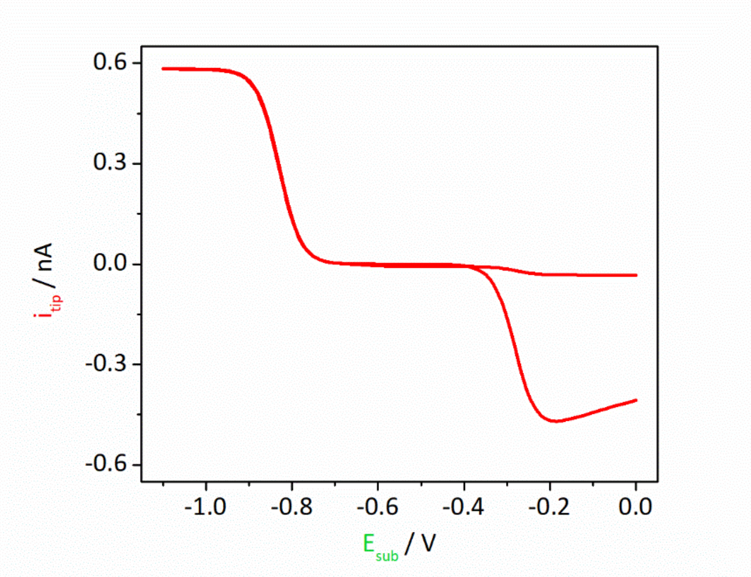

Figure S19. Simulation of experiment shown in Figure 4c. CV performed at the substrate electrode while E_{tip} is kept at a potential of –0.6 V. Conditions: C(**1**) = 380 μM, C(**3**$^{2+}$) = 240 μM, d = 4 μm.

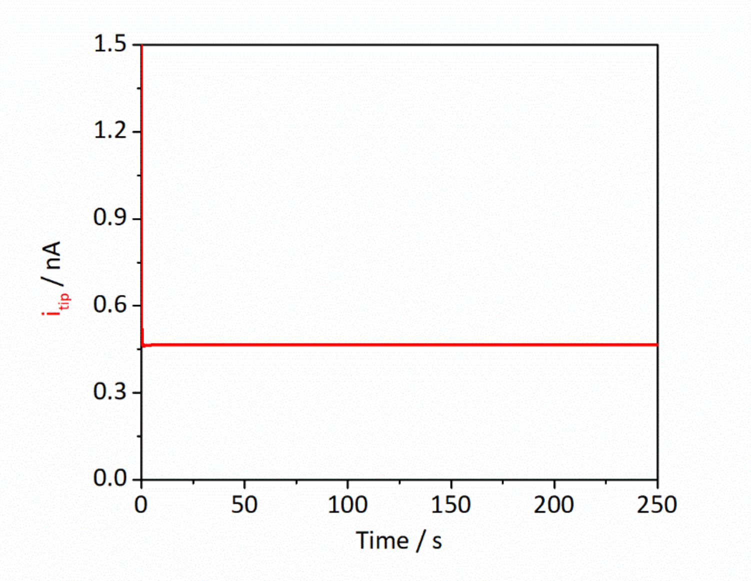

Figure S20. Simulation of the amperometric experiment reported in Figure 5a in which E_{sub} = –0.8 V and E_{tip} = 0 V. Conditions: C(**1**) = 380 μM, C(**2**$^{2+}$) = 240 μM, d = 25 μm.

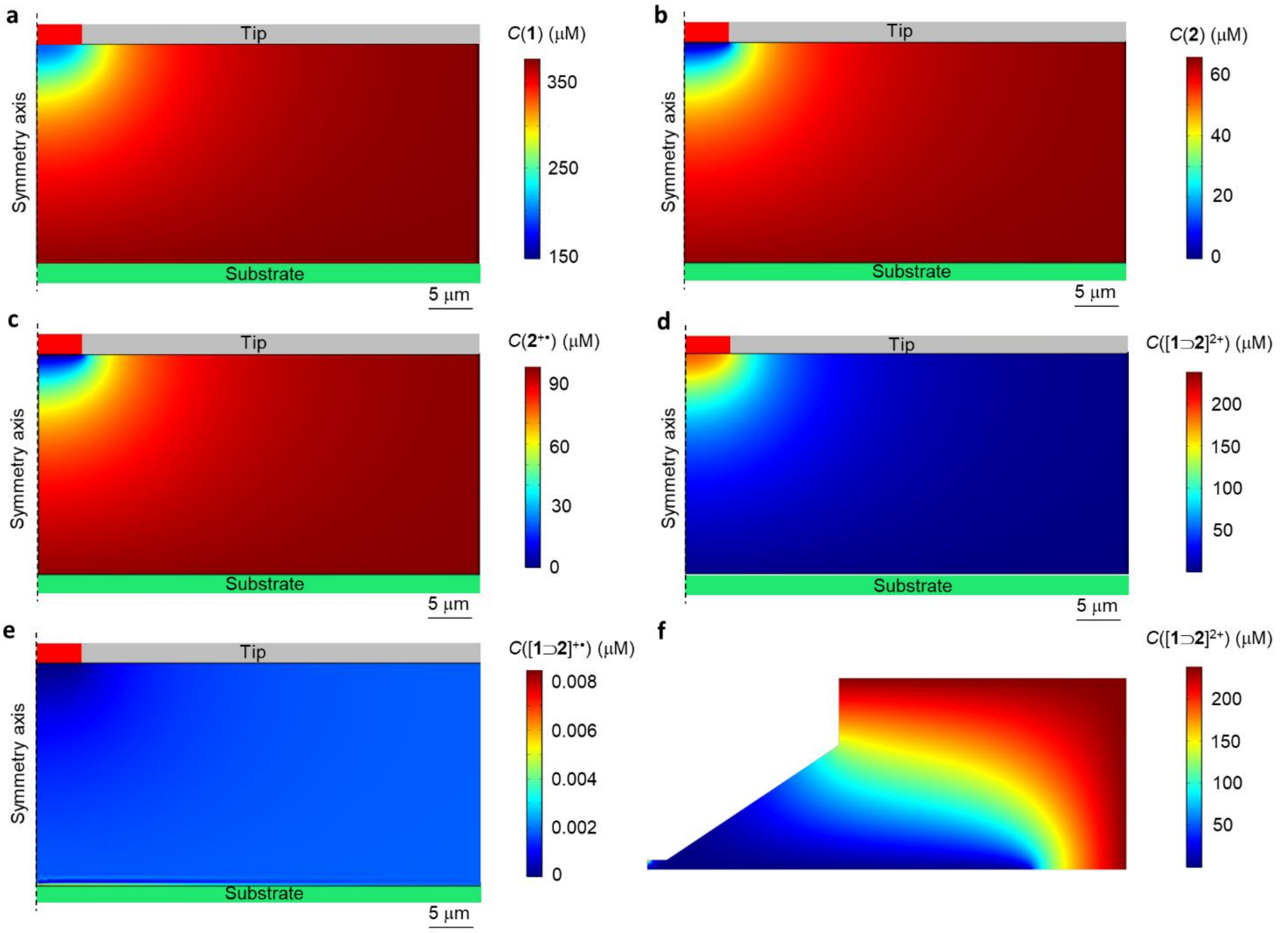

Figure S21. Stationary concentrations of (a) **1**, (b) **2**, (c) $\mathbf{2}^{+\bullet}$, (d) $[\mathbf{1}\supset\mathbf{2}]^{2+}$, and (e) $[\mathbf{1}\supset\mathbf{2}]^{+\bullet}$, obtained from the simulation of the amperometric experiment in which electrode potentials are fixed at E_{sub} = –0.8 V and E_{tip} = 0 V. The shown region of space corresponds to the rectangular section comprised between tip and substrate shown in Figure S12b. Panel f reports the full simulated map for $[\mathbf{1}\supset\mathbf{2}]^{2+}$, to highlight boundary conditions. Conditions: C(**1**) = 380 μM, $C(\mathbf{2}^{2+})$ = 240 μM, d = 25 μm, t = 150 s (Figure S20).

Since reduction and dethreading afford $\mathbf{2}^{+\bullet}$, a species diffusing faster than $[\mathbf{1}\supset\mathbf{2}]^{2+}$, diffusion of $\mathbf{2}^{+\bullet}$ away from the electrode is not completely counterbalanced by the slower diffusion of $[\mathbf{1}\supset\mathbf{2}]^{2+}$ towards the substrate electrode, leading to a non-equilibrium steady-state in which the local concentration of all species containing 1,10-dipentyl-4,4'-bipyridinium is lower than its bulk concentration (imposed as boundary condition). This effect is little when reduction occurs at the smaller tip electrode, but significant when reduction occurs at the larger substrate electrode. The origin of this effect was confirmed by performing amperometric simulations using the same diffusion coefficient for all species containing 1,10-dipentyl-4,4'-bipyridinium. In these cases, the total concentration of 1,10-dipentyl-4,4'-bipyridinium remains uniform and equal to the bulk concentration throughout the whole simulated volume.

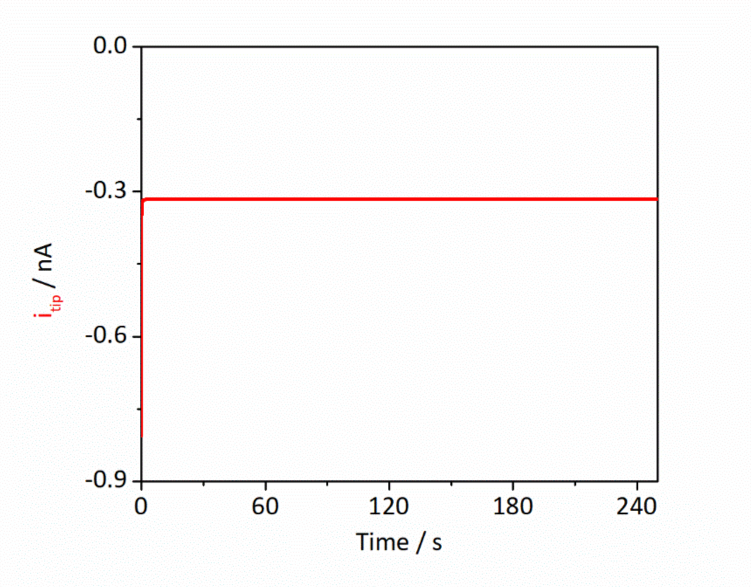

Figure S22. Simulation of the amperometric experiment reported in Figure S10 in which E_{sub} = 0 V and E_{tip} = –0.8 V. Conditions: C(**1**) = 380 μM, C(**2**$^{2+}$) = 240 μM, d = 25 μm.

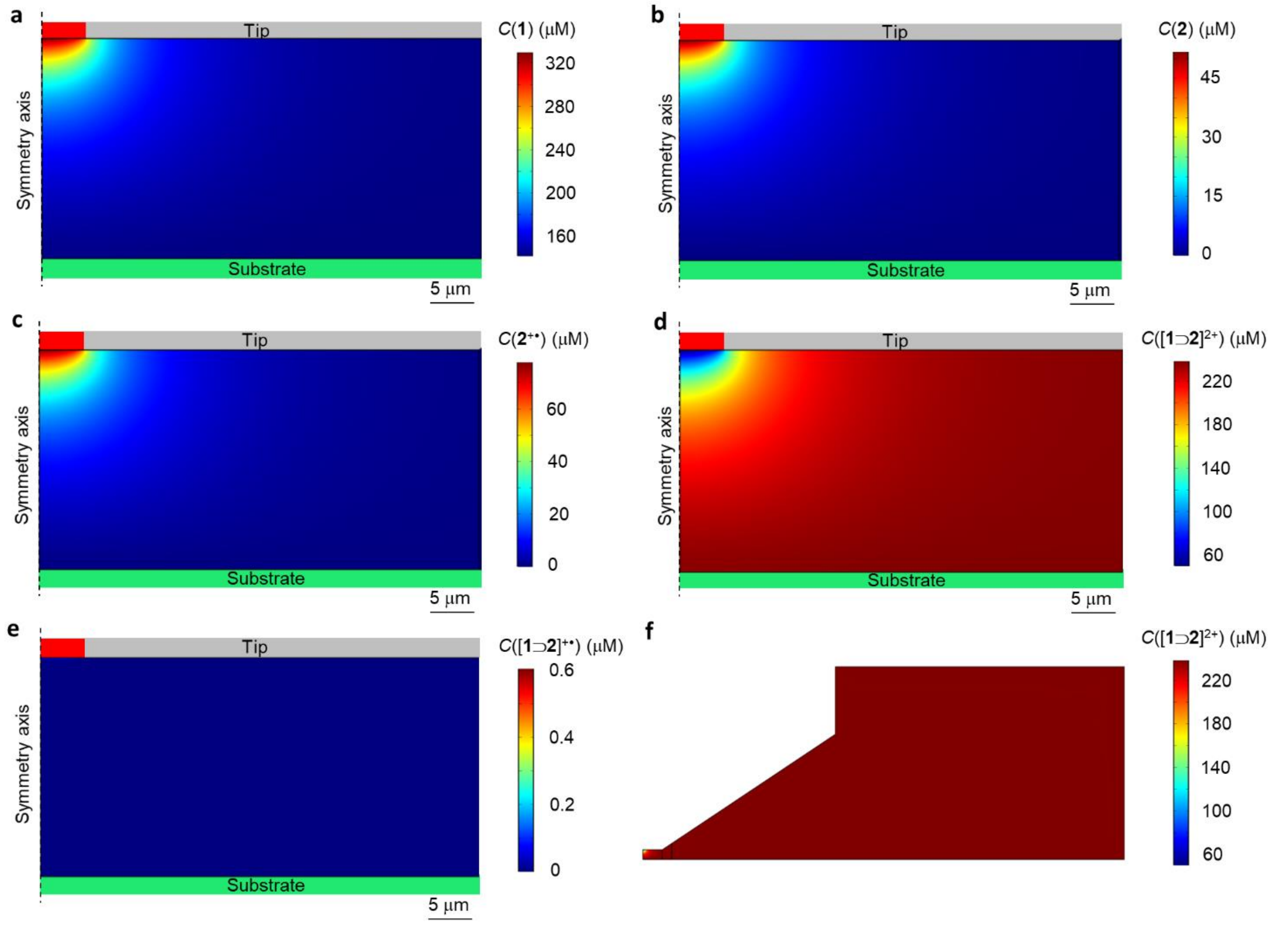

Figure S23. Stationary concentrations of (a) **1**, (b) **2**, (c) **2**$^{+\bullet}$, (d) [**1**⊃**2**]$^{2+}$, and (e) [**1**⊃**2**]$^{+\bullet}$, obtained from the simulation of the amperometric experiment in which electrode potentials are fixed at E_{sub} = 0 V and E_{tip} = –0.8 V. The shown region of space corresponds to the rectangular section comprised between tip and substrate shown in Figure S12b. Panel f reports the full simulated map for [**1**⊃**2**]$^{2+}$, to highlight boundary conditions. Conditions: C(**1**) = 380 μM, C(**2**$^{2+}$) = 240 μM, d = 25 μm, t = 150 s (Figure S22).

9. Insights on system performance

Energetic considerations are referred to the amperometric simulations reported in Figure S20 (E_{tip} = 0 V) and Figure S22 (E_{tip} = –0.8 V). Values have been evaluated at the stationary state (t = 150 s), at T = 298 K. Integrations have been performed directly in COMSOL.

Chemical rates and net fluxes were defined as follows:

$$v^{B}_{ox} = k^{B}_{ox}\, C(\mathbf{1})\, C(\mathbf{2}^{2+}) \quad (12)$$

$$v^{A}_{ox} = k^{A}_{ox}\, C([\mathbf{1}\supset\mathbf{2}]^{2+}) \quad (13)$$

$$v_{ox} = v^{B}_{ox} - v^{A}_{ox} \quad (14)$$

$$v^{B}_{red} = k^{B}_{red}\, C(\mathbf{1})\, C(\mathbf{2}^{+\bullet}) \quad (15)$$

$$v^{A}_{red} = k^{A}_{red}\, C([\mathbf{1}\supset\mathbf{2}]^{+\bullet}) \quad (16)$$

$$v_{red} = v^{B}_{red} - v^{A}_{red} \quad (17)$$

The dissipated power (Σ) was calculated as follows:[13]

$$\Sigma_{ox} = R\, T\, (v_{ox}) \ln (v^{B}_{ox} / v^{A}_{ox}) \quad (18)$$

$$\Sigma_{red} = R\, T\, (v_{red}) \ln (v^{B}_{red} / v^{A}_{red}) \quad (19)$$

$$\Sigma = \Sigma_{ox} + \Sigma_{red} \quad (20)$$

The values of Σ are reported in Figure S24. The total dissipated power is obtained upon integration of the dissipated power over a given volume. The maps of dissipated power (Figure S24) indicate that dissipation processes occur in proximity of the electrodes, largely within the first 25 μm from the symmetry axis. Therefore Σ_{ox} and Σ_{red} were integrated over a cylindric volume with radius three times larger than this distance, equal to 75 μm (all the space shown in Figure S24), affording $\Sigma^{V} = \Sigma^{V}_{ox} + \Sigma^{V}_{red}$. Integration over the entire volume would not be representative due to reactions involving bulk species, as occurring in real experiments. Integrating over a slightly larger or smaller cylinder (e.g. 70 or 100 μm) afforded identical results, confirming that at ca. 75 μm all chemical and electrochemical processes are negligible. Accordingly, also the net assembly rates v_{ox} and v_{red} were significantly different from 0 only within this volume, which was employed to integrate in space the assembly rates, to obtain the resulting v^{V}_{ox} and v^{V}_{red}.
Taking this volume as reference was further corroborated by the fact that in the same portion of space $i_{sub} = -i_{tip}$ in both amperometric experiments within <5% error, which is in good agreement with the experimental collection efficiency.

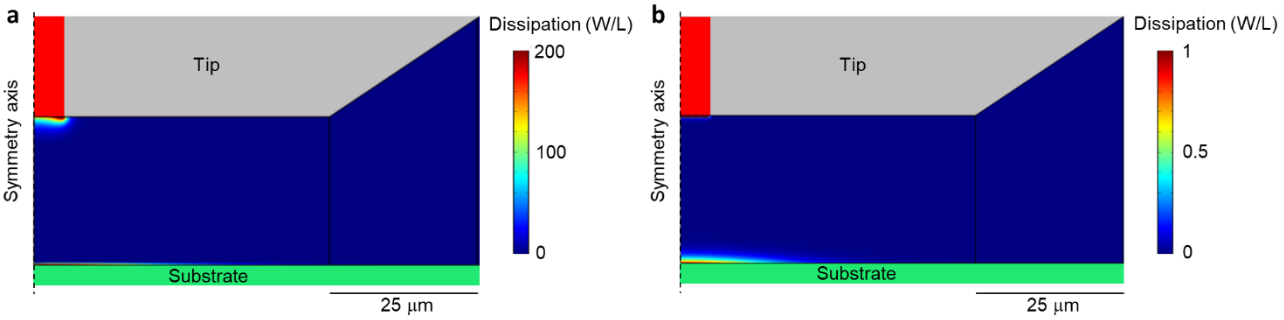

Figure S24. Stationary-state dissipation (Σ) associated with the self-assembly reaction of **1** with $\mathbf{2}^{2+}$ and $\mathbf{2}^{+\bullet}$, obtained from a simulation of an amperometric experiment in which (a) electrode potentials are fixed at E_{sub} = 0 V and E_{tip} = –0.8 V or (b) E_{sub} = –0.8 V and E_{tip} = 0 V. Conditions: $C(\mathbf{1})$ = 380 μM, $C(\mathbf{2}^{2+})$ = 240 μM, d = 25 μm, t = 150 s. The scale of panel b has been selected to highlight the dissipation close to the substrate, while most of the dissipation occurs close to the tip, where values up to ca. 10^4 W/L are observed. Selecting a different scale does not improve the visualization of such a high dissipation in close proximity of the tip.

Dynamic properties. In the simulation performed at E_{sub} = –0.8 V and E_{tip} = 0 V, i_{tip} = 0.465 nA (Figure S20) and v^{V}_{ox} = 3.4×10^{-6} nmol s^{-1} = 3.4 fmol s^{-1}.

In 1 s the moles of electron injected in the system are:

$n(e^{-})$ = 0.465 nA × 1 s / 96485 s A mol^{-1} = 4.82×10^{-6} nmol (21)

It follows that the average number of electrons required to complete one cycle of threading and dethreading are:

$N(e^{-})$ = 4.82×10^{-6} nmol / 3.4×10^{-6} nmol = 1.4 (22)

The need for more than one electron to complete a cycle is ascribed to the concomitant reduction of **2**$^{+\bullet}$ to **2**, which occurs at –0.81 V, a potential close to the operating one, acting as an unproductive side-reaction.

Integration of the concentration of all species comprising the axle in the cylindric volume considered in proximity of the tip (*vide supra*) afforded a value of 86.9 fmol of axles (in any form) in the considered domain. Thus, the time required for all these species to undergo a cycle is:

t_{cycle} = 86.9 fmol / 3.4 fmol s^{-1} = 26 s (23)

In the simulation performed at E_{sub} = 0 V and E_{tip} = –0.8 V, i_{tip} = –0.315 nA (Figure S22) and v^{V}_{ox} = 2.2×10^{-6} nmol s^{-1} = 2.2 fmol s^{-1}. Following an identical mathematical procedure, it was calculated that under this setup 1.5 electrons are required to complete one cycle, and 55 s are required for all the axles (127 fmol) in proximity to the cycle to complete one cycle.

Efficiency. In the simulation performed at E_{sub} = –0.8 V and E_{tip} = 0 V, Σ^{V}_{ox} = 2.26×10^{-2} nW and Σ^{V}_{red} = 2.10×10^{-2} nW. The value of Σ^{V}_{red} is subject to slight changes (e.g. by 20%) upon further refining of the mesh. The reason for the observed behavior is tentatively ascribed to numerical difficulties to accurately reproduce such a fast process involving species in low concentration (see Figure S21e). Refining the mesh leaves unaltered the values of Σ^{V}_{ox} (and i_{tip}). For this reason, we calculated the dissipation only considering the contribute of Σ^{V}_{ox} to the total dissipation. Since the dissipation can only be larger than zero, the resulting efficiency value is underestimated, i.e., considering the contribute of Σ^{V}_{red} the efficiency would be even higher.

We estimate an experimental lower bound for the energy injected in the system considering that most of the current is sustained between the substrate electrode and the counter electrode. i_{sub} = 3.5 µA is an experimentally accessible value, while $E_{counter}$ is assumed being 2.0 V considering the electrochemical window of CH_2Cl_2. Therefore, the energy injected in the whole system in 1 s is:

W_{in} = 3.5 µC × 2.8 V = 9.8 µJ (24)

The energy conversion efficiency is thus:

$\eta = \Sigma^{V} \times \Delta t / W_{in}$ = 2.26×10^{-2} nW × 1 s / 9.8 µJ = 2.3×10^{-6} (25)

Which represents a lower bound for the estimated efficiency. This estimation reflects the capability of employed experimental setup, and is essentially determined by the unproductive energy loss taking place between the substrate and counter electrodes.

To have an estimation of the efficiency that can be compared with that of other light- and chemically-driven motors for which a detailed thermodynamic treatment is available, we need to apply the same approximations, excluding the background energy loss. Therefore, the efficiency is recalculated considering i_{tip} = 0.465 nA and the potential difference between tip and substrate electrodes.

The energy injected in the system in 1 s is:

W_{in} = 0.465 nC × 0.8 V = 0.375 nJ (26)

The energy conversion efficiency is thus:

$\eta = \Sigma^{V} \times \Delta t / W_{in}$ = 2.26×10^{-2} nW × 1 s / 0.375 nJ = 0.06 (27)

In the simulation performed with E_{sub} = 0 V and E_{tip} = –0.8 V, Σ^{V}_{ox} = 8.3×10^{-4} nW and Σ^{V}_{red} = 2.28×10^{-2} nW.

The total dissipation is thus:

$$\Sigma^{V} = \Sigma^{V}_{ox} + \Sigma^{V}_{red} = 8.3\times10^{-4}\ \text{nW} + 2.20\times10^{-2}\ \text{nW} = 2.28\times10^{-2}\ \text{nW} \quad (28)$$

Following a mathematical procedure identical to the previous case, an efficiency η = 0.09 is obtained.

We note here that the thermodynamic efficiency considered herein is different from the efficiency considered by other authors in related contexts.[14,15,16] Systems for which thermodynamic efficiency has been calculated are reported in Table S7.

Table S7. Thermodynamic efficiency of autonomous systems for which such efficiency has been reported.

System	Thermodynamic efficiency
Chemically-driven rotary catenane[17,18]	10^{-8}
Light-driven pump[19,20]	≤3×10^{-4}
Light-driven pump[21]	≤ 4×10^{-3}

Operation mechanism. To validate the operation of an energy ratchet mechanism we took as reference the simulation performed at E_{sub} = 0 V and E_{tip} = –0.8 V (Figure S22), in which v^{V}_{ox} = 2.2 fmol s^{-1} was observed. The stability of oxidized and reduced species was inverted by exchanging the rate constants (e.g. k^{B}_{ox} = 2.3×10^{3} (m^{3} mol^{-1} s^{-1}) was employed here as k^{B}_{red}). Consequently, reduction potentials were also inverted to satisfy constraints imposed by microscopic reversibility. Using the inverted stabilities, a current of v^{V}_{ox} = –3.3 fmol s^{-1} was observed at the steady state.

10. References

1. Arduini, A.; Bussolati, R.; Credi, A.; Secchi, A.; Silvi, S.; Semeraro, M.; Venturi, M. Toward Directionally Controlled Molecular Motions and Kinetic Intra- and Intermolecular Self-Sorting: Threading Processes of Nonsymmetric Wheel and Axle Components. *J. Am. Chem. Soc.* **2013**, *135*, 9924–9930.
2. Binstead, R. A. *SPECFIT*, Spectrum Software Associates, Chapel Hill, USA, **1996**.
3. Lefrou, C. A unified new analytical approximation for positive feedback currents with a microdisk SECM tip. *J. Electroanal. Chem.* **2006**, 592, 103–112.

4 Valenti, G.; Bardini, L.; Bonazzi, D.; Rapino, S.; Marcaccio, M.; Paolucci, F. Creation of Reactive Micro Patterns on Silicon by Scanning Electrochemical Microscopy. *J. Phys. Chem. C* **2010**, 114, 50, 22165–22170.

5 Daviddi, E.; Oleinick, A.; Svir, I.; Valenti, G.; Paolucci, F.; Amatore, C. Theory and Simulation for Optimising Electrogenerated Chemiluminescence from Tris(2,2′-bipyridine)-ruthenium(II)-Doped Silica Nanoparticles and Tripropylamine. *ChemElectroChem* **2017**, 4, 1719–1730.

6. Zanello, P.; Fabrizi de Biani, F.; Nervi, C. *Inorganic Electrochemistry: Theory, Practice and Application*, RSC Publishing: Cambridge, 2012.
7. Janisch, J.; Ruff, A.; Speiser, B.; Wolff, C.; Zigelli, J.; Benthin, S.; Feldmann, V.; Mayer, H. A. Consistent Diffusion Coefficients of Ferrocene in Some Non-Aqueous Solvents: Electrochemical Simultaneous Determination Together with Electrode Sizes and Comparison to Pulse-Gradient Spin-Echo NMR Results. *J. Solid State Electrochem.* **2011**, 15, 2083–2094.
8. Evans, R.; Dal Poggetto, G.; Nilsson, M.; Morris, G. A. Improving the Interpretation of Small Molecule Diffusion Coefficients. *Anal. Chem.* **2018**, *90*, 3987−3994.
9. Polson, A. The Some Aspects of Diffusion in Solution and a Definition of a Colloidal Particle. *J. Phys. Chem.* **1950**, *54*, 649–652.

10 Credi, A.; Dumas, S.; Silvi, S.; Venturi, M.; Arduini, A.; Pochini, A.; Secchi, A. Viologen-Calix[6]arene Pseudorotaxanes. Ion-Pair Recognition and Threading/Dethreading Molecular Motions. *J. Org. Chem.* **2004**, *69*, 5881–5887.

11 Semeraro, M.; Secchi, A.; Silvi, S.; Venturi, M.; Arduini, A.; Credi, A. Photoinduced electron transfer from $[Ru(bpy)_3]^{2+}$ to a calix[6]arene-encapsulated viologen electron acceptor. *Inorganica Chim. Acta* **2014**, 417, 258–262.

12 Cook, S. K.; Horrocks, B. R. Heterogeneous Electron-Transfer Rates for the Reduction of Viologen Derivatives at Platinum and Bismuth Electrodes in Acetonitrile. *ChemElectroChem* **2017**, *4*, 320–331.

13 Rao, R.; Esposito, M. Nonequilibrium Thermodynamics of Chemical Reaction Networks: Wisdom from Stochastic Thermodynamics. *Phys. Rev. X* **2016**, *6*, 041064.

14 Conyard, J.; Cnossen, A.; Browne, W. R.; Feringa, B. L., Meech, S. R. Chemically Optimizing Operational Efficiency of Molecular Rotary Motors. *J. Am. Chem. Soc.* **2014**, 136, 27, 9692–9700.

15 Larson, A. M.; Balema, T. A.; Zahl, P.; Schilling, A. C.; Stacchiola, D. J.; Sykes, C. H. Hypothetical Efficiency of Electrical to Mechanical Energy Transfer during Individual Stochastic Molecular Switching Events. *ACS Nano* **2020**, *14*, 16558–16564.

16 Borsley, S.; Leigh, D. A.; Roberts, B. M. W.; Vitorica-Yrezabal, I. J. Tuning the force, speed, and efficiency of an autonomous chemically fuelled information ratchet. *J. Am. Chem. Soc.* **2022**, 144, 17241–17248.

17 Wilson, M. R.; Solà, J.; Carlone, A.; Goldup, S. M.; Lebrasseur, N.; Leigh, D. A. An autonomous chemically fuelled small-molecule motor. *Nature* **2016**, *534*, 235–240.

18 Amano, S.; Esposito, M.; Kreidt, E.; Leigh, D. A.; Penocchio, E.; Roberts, B. M. W. Insights from an information thermodynamics analysis of a synthetic molecular motor. *Nat. Chem.* **2021**, *14*, 530–537.

19 Ragazzon, G.; Baroncini, M.; Silvi, S.; Venturi, M; Credi, A. Light-powered autonomous and directional molecular motion of a dissipative self-assembling system, *Nat. Nanotechnol.* **2015**, *10*, 70–75.

20 Penocchio, E.; Avanzini, F.; Esposito, M. Information thermodynamics for deterministic chemical reaction networks. *J. Chem. Phys.* **2022**, *157*, 034110.

21 Corra, S.; Bakic, M. T.; Groppi, J.; Baroncini, M.; Silvi, S.; Penocchio, E.; Esposito, M.; Credi, A. Kinetic and energetic insights into the dissipative non-equilibrium operation of an autonomous light-powered supramolecular pump. *Nat. Nanotechnol.* **2022**, *17*, 746–751.
